# Electromagnetic chirality: from fundamentals to nontraditional chiroptical phenomena

**DOI:** 10.1038/s41377-020-00367-8

**Published:** 2020-09-02

**Authors:** Jungho Mun, Minkyung Kim, Younghwan Yang, Trevon Badloe, Jincheng Ni, Yang Chen, Cheng-Wei Qiu, Junsuk Rho

**Affiliations:** 1 Department of Chemical Engineering, Pohang University of Science and Technology, Pohang, 37673 Korea; 2 Department of Mechanical Engineering, Pohang University of Science and Technology, Pohang, 37673 Korea; 3 Department of Electrical and Computer Engineering, National University of Singapore, Singapore, 117583 Singapore

**Keywords:** Nanophotonics and plasmonics, Metamaterials, Nanoparticles, Sub-wavelength optics

## Abstract

Chirality arises universally across many different fields. Recent advancements in artificial nanomaterials have demonstrated chiroptical responses that far exceed those found in natural materials. Chiroptical phenomena are complicated processes that involve transitions between states with opposite parities, and solid interpretations of these observations are yet to be clearly provided. In this review, we present a comprehensive overview of the theoretical aspects of chirality in light, nanostructures, and nanosystems and their chiroptical interactions. Descriptions of observed chiroptical phenomena based on these fundamentals are intensively discussed. We start with the strong intrinsic and extrinsic chirality in plasmonic nanoparticle systems, followed by enantioselective sensing and optical manipulation, and then conclude with orbital angular momentum-dependent responses. This review will be helpful for understanding the mechanisms behind chiroptical phenomena based on underlying chiral properties and useful for interpreting chiroptical systems for further studies.

## Towards an extended understanding of chiroptical phenomena

Strengthening the theoretical understanding of chirality is necessary for developing applications based on its phenomena. Junsuk Rho of Korea’s Pohang University of Science and Technology (POSTECH) reviewed with colleagues the theoretical aspects of chirality, a symmetry property that describes mirror-image objects or systems that cannot be superimposed. Chiral materials have attracted much attention due to their interesting interactions. Scientists are familiar with how geometrically chiral objects and systems interact with light. However, such ‘chiroptical effects’ can also be found in achiral systems, Rho and his colleagues explain. Also, globally achiral light can be locally chiral near nanostructures. Scientists need to extend their concepts and theoretical understandings of chiroptical systems in order to be able to further develop applications based on their phenomena, such as in metamaterials, sensing, spintronics and stereochemistry.

## Introduction

Chiral objects are widespread in nature, with some examples being DNA and protein. A chiral object or system is defined as one for which the structure and its mirror image (enantiomer) are not superimposable. Although most of their physical properties are the same, a chiral object and its enantiomer may exhibit different responses. One can directly determine whether an object is chiral from its geometry alone; in other words, geometric chirality is a qualitative, binary property. In contrast, chiroptical effects are quantitatively measurable. Some commonly measured chiroptical phenomena include optical rotatory dispersion (ORD), measured by the degree of polarization rotation, and circular dichroism (CD), the difference in the absorption of left-circularly polarized (LCP) and right-circularly polarized (RCP) light. However, to the best of our knowledge, geometric chirality cannot be quantified, and no universal rule exists for predicting the relationship between the geometric chirality of an object and its chiroptical responses without the use of numerical tools.

**Fig. 1:**
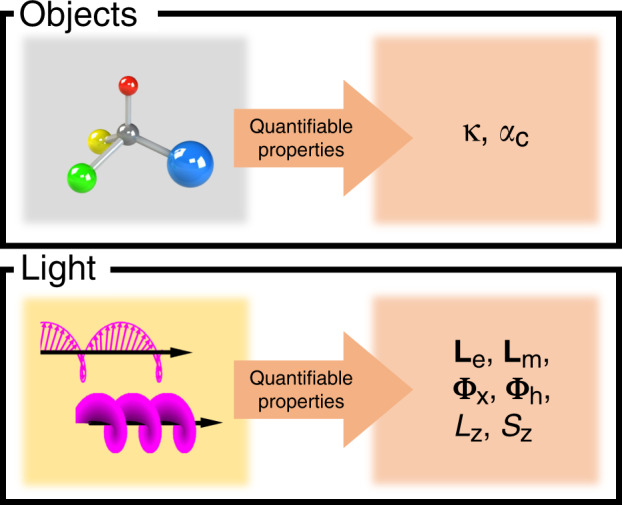
Overview of the quantifiable properties of chirality. The chirality of objects can be described quantitively in terms of the chirality parameter (*κ*) and chiral polarizability tensor (*α*_c_). Similarly, for light, the spin-density fluxes (**L**_e_ and **L**_m_), optical helicity fluxes (${\mathbf{\Phi}}_\chi$ and ${\mathbf{\Phi}}_h$), and orbital and spin angular momenta (*L*_*z*_ and *S*_*z*_) give it quantifiable chiral properties

Although chirality itself is a qualitative property, chiroptical systems can be quantitatively modeled by chiroptical parameters, as shown in Fig. 1. Light cannot be chiral in the conventional sense, as it possesses no geometry, but the chirality of light can be defined with certain parameters. For LCP or RCP light, chiral properties arise from the rotating electric and magnetic vector fields as the wave propagates. Light can also carry orbital angular momentum, which creates a helical geometry on the wavefront. The amount of chirality that light carries can be expressed in terms of chiroptical parameters such as the spin-density fluxes (**L**_e_ and **L**_m_), optical helicity fluxes (${\mathbf{\Phi}}_\chi$ and ${\mathbf{\Phi}}_{h}$), and orbital and spin angular momenta (*L*_*z*_ and *S*_*z*_). These parameters allow us to define chiral light. Similarly, the chirality of an object can be quantified in terms of the chirality parameter (*κ*) and chiral polarizability tensor (*α*_c_).

To understand these chiroptical parameters, we should first consider parity symmetry ${\mathcal{P}}$ and time-reversal symmetry ${\mathcal{T}}$. The ${\mathcal{P}}$ operator transforms physical quantities as **x** → −**x**, *t* → *t*, **E** → −**E**, and **H** → **H**, whereas the ${\mathcal{T}}$ operator transforms them as **x** → **x**, *t* → −*t*, **E** → **E**, and **H** → −**H**, where **x** is a space coordinate, *t* is time, **E** is the electric field, and **H** is the magnetic field. Since geometric chirality is given by broken space-parity symmetry, the chiroptical observables of interest are parity-odd and time-even pseudoscalars^1^. This symmetry-based framework provides a guideline to determine which parameters contribute to chiroptical phenomena^2^. Chiroptical phenomena can also be observed in nonreciprocal systems (e.g., Faraday rotation), and such phenomena are termed false chirality^1^. Here, we focus on the true chirality of reciprocal systems.

Chirality and chiral phenomena associated with electromagnetic (EM) waves have recently been covered in many review papers, mostly focused on various designs and fabrication methods of chiral metamaterial systems to increase chiral responses^3–6^. Here, we concentrate on the theoretical framework that describes chiral systems and chiral EM fields and on advances in the quantification of EM field chirality and introduce examples of exploiting chiral light–matter interactions for specific applications. We aim to present the importance of understanding the properties that give light chirality, light–matter interactions, and the resultant chiroptical phenomena in applications such as chiral sensing.

In this review, we present theoretical frameworks for describing continuous chiral media, chiral particle systems, and chiral EM fields. We continue by explaining two chiroptical phenomena: chiroptical manipulation and the chiral light–matter interactions produced by orbital angular momentum. We conclude by summarizing the quantifiable chiral parameters and finally provide our perspective on future directions for research in the field of chirality.

## Strong artificial structural chirality

Natural materials generally exhibit very weak chiroptical properties due to the large mismatch between their atomic feature sizes and optical wavelengths; however, strong handedness-dependent responses have been observed for structured materials such as the scarab beetle *Chrysina gloriosa*, which selectively reflects LCP light^7^, and for artificially engineered chiral structures. Several review papers^4,6,8,9^ have recently covered the experimental realization of these artificial chiral plasmonic systems. In this section, we discuss how the EM chirality of continuous chiroptical media and microscopic chiral particles are modeled and how to interpret chiroptical phenomena using the framework.

### Continuous chiroptical media: chiral metamaterials

First, we briefly discuss light propagation in a continuous chiroptical medium, which has been extensively studied in the context of chiral molecular media^10^ and chiral metamaterials^3,11,12^. By introducing the chirality parameter *κ*, a reciprocal isotropic chiral medium can be modeled by the constitutive relations^13^:

1 $$\left( {\begin{array}{*{20}{c}} {{\mathbf{D}}/{\it{\epsilon }}_0} \\ {c{\mathbf{B}}} \end{array}} \right) = \left( {\begin{array}{*{20}{c}} {{\it{\epsilon }}_{\mathrm{r}}} & {i\kappa } \\ { - i\kappa } & {\mu _{\mathrm{r}}} \end{array}} \right)\left( {\begin{array}{*{20}{c}} {\mathbf{E}} \\ {\eta _0{\mathbf{H}}} \end{array}} \right)$$

where **D** is the electric displacement field, **B** is the magnetic induction field, *c* is the speed of light, *ϵ*_0_ is the vacuum permittivity, *ϵ*_r_ is the relative permittivity, *μ*_r_ is the relative permeability, *κ* is the chirality parameter, and *η*_0_ = (*μ*_0_/*ϵ*_0_)^1/2^ is the vacuum wave impedance. Note that field quantities are expressed using SI units throughout this review. The refractive indices of LCP and RCP light in this chiral medium are *n*_±_ = (*ϵ*_r_*μ*_r_)^1/2^ ± *κ*. Since *κ* relates two quantities with opposite parities (i.e., **D** and **H**; **B** and **E**), it is parity-odd and therefore contributes to chiroptical phenomena. The ORD and CD of light propagating in a homogeneous chiral medium are related to *κ* as follows: ORD ∝ Re(*κ*)*l* and CD ∝ Im(*κ*)*l*, where *l* is the optical path length. The real and imaginary parts of *κ* (CD and ORD) are connected by the Kramers–Kronig relationship. This description (Eq. 1) has been widely used to study EM phenomena involving chiral media to predict photonic topological materials^14^ and negative refractive indices^15^.

### Small chiral particles: dipole approximation and polarizability

**Fig. 2:**
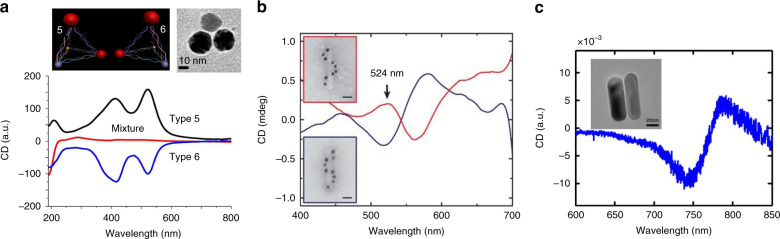
Examples of experimentally realized small plasmonic chiral particles and their CD spectra. **a** Asymmetric pyramidal and **b** helical assemblies of plasmonic nanospheres, and **c** a dimer composed of twisted nanorods. **a** Adapted with permission from ref. ^17^, Copyright 2012, ACS. **b** Adapted with permission from ref. ^18^, copyright 2012, NPG. **c** Adapted with permission from ref. ^19^, copyright 2013, NPG

Effectively continuous chiral media can be achieved by using subwavelength chiral particles as meta-atoms, but small scattering systems (e.g., chiral molecules and subwavelength nanoparticles) usually exhibit very weak chiral responses because subwavelength particles are generally dominated by the electric dipole response, and chiroptical responses originate from transitions between modes with opposite parities^16^. Surprisingly, strong chiroptical responses far exceeding those in naturally occurring chiral systems have been observed for subwavelength plasmonic chiral systems, including asymmetric tetrahedral^17^ and helical assemblies of nanospheres^18^ and twisted nanorod dimers^19^ (Fig. 2). Their chiroptical effects are observed near their localized surface plasmon resonance wavelengths in the visible regime and are reversed with a change in their geometric handedness. Such colloidal chiral particles are typically fabricated using the DNA self-assembly method whereby achiral plasmonic nanoparticles are assembled into chiral systems. Although the particles are highly anisotropic, they are randomly dispersed and therefore exhibit orientation-averaged responses.

We now discuss the microscopic description of chiral particles and their strong chiroptical phenomena. The dipole approximation used to intuitively explain the optical responses of small particles also applies to small chiral particles. A point-like chiral system can be described by the dynamic polarizability tensor, which is a transition matrix that linearly maps the incident (excitation) field to the induced moments as:

2 $$\left( {\begin{array}{*{20}{c}} {{\mathbf{p}}/{\it{\epsilon }}} \\ {\eta {\mathbf{m}}} \end{array}} \right) = \left( {\begin{array}{*{20}{c}} {\alpha _{\mathrm{e}}} & {i\alpha _{\mathrm{c}}} \\ { - i\alpha _{\mathrm{c}}^ \top } & {\alpha _{\mathrm{m}}} \end{array}} \right)\left( {\begin{array}{*{20}{c}} {\mathbf{E}} \\ {\eta {\mathbf{H}}} \end{array}} \right)$$

where **p** is the induced electric dipole (ED) moment, **m** is the induced magnetic dipole (MD) moment, *ϵ* is the permittivity of the host medium and *η* is its wave impedance. The polarizability tensors *α*_e_, *α*_m_, and *α*_c_ have dimensions of volume and are generally 3 × 3 matrices. Under the space-parity operation, **p** and **m** transform as ${\mathcal{P}}$[**p**] → −**p** and ${\mathcal{P}}$[**m**] → **m**. *α*_c_ relates two quantities with opposite parities (i.e., **E** and **m**; **H** and **p**), so it is parity-odd and contributes to chiroptical phenomena. In some of the literature, polarizabilities are defined using **B** instead of **H**, but this does not affect the main results. In the quasi-static limit, the polarizabilities of a chiral sphere of radius *R* are^13,20^:

3 $$\begin{array}{l}\alpha _{\mathrm{e}} = 4\pi R^3\frac{{\left( {{\it{\epsilon }}_{\mathrm{r}} \,-\, 1} \right)\left( {\mu _{\mathrm{r}} \,+\, 2} \right) \,-\, \kappa ^2}}{{\left( {{\it{\epsilon }}_{\mathrm{r}} \,+\, 2} \right)\left( {\mu _{\mathrm{r}} \,+\, 2} \right) \,-\, \kappa ^2}}\\ \alpha _{\mathrm{m}} = 4\pi R^3\frac{{\left( {{\it{\epsilon }}_{\mathrm{r}} \,+\, 2} \right)\left( {\mu _{\mathrm{r}} \,-\, 1} \right) \,-\, \kappa ^2}}{{\left( {{\it{\epsilon }}_{\mathrm{r}} \,+\, 2} \right)\left( {\mu _{\mathrm{r}} \,+\, 2} \right) \,-\, \kappa ^2}}\\ \alpha _{\mathrm{c}} = 4\pi R^3\frac{{3\kappa }}{{\left( {{\it{\epsilon }}_{\mathrm{r}} \,+\, 2} \right)\left( {\mu _{\mathrm{r}} \,+\, 2} \right) \,-\, \kappa ^2}}\end{array}$$

These dynamic polarizabilities have been widely used to describe small chiral particles or molecules to study chiral optomechanical^20,21^ and scattering processes^22–25^. The dynamic polarizability tensors of general nonspherical particles can also be retrieved^26,27^. For a deeper discussion, please refer to ref. ^28^.

**Fig. 3:**
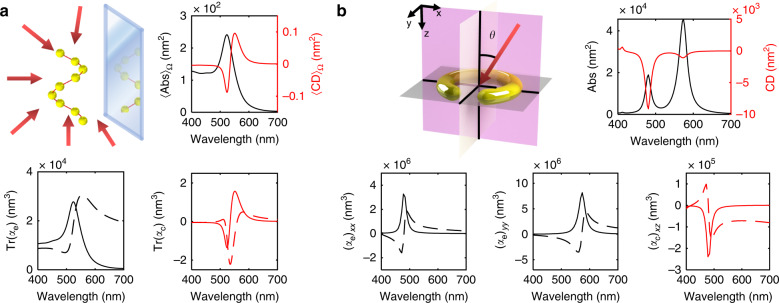
Multipole-based approach to describe intrinsic chirality and extrinsic chirality. **a** Intrinsic and **b** extrinsic chirality of subwavelength plasmonic chiral nanoparticles. **a** Schematic, 〈Abs〉_Ω_ and 〈CD〉_Ω_, Tr(*α*_e_), and Tr(*α*_c_) of a helical assembly of nine Au nanospheres with a major radius of 17nm, a minor radius of 5nm, and a helical pitch of 57nm. **b** Schematic, Abs and CD, (*α*_e_)_*xx*_, (*α*_e_)_*yy*_, and (*α*_c_)_*xz*_ of a Ag SRR with a major radius of 30nm, a minor radius of 10nm, and a split angle of 60°. Solid lines represent the imaginary part and dashed lines represent the real part of *α*_e_ and *α*_c_. Panel **a** was calculated using Mie theory, and panel **b** was calculated using the commercial FEM solver COMSOL Multiphysics 5.4

Strong chiroptical effects from small plasmonic chiral particles are allowed due to the interplay between the ED and MD^29^, i.e., the magnetoelectric polarizability *α*_c_. High refractive index dielectric nanostructures also rely on this intermode transition^30^. Using exemplary plasmonic particles, we show how chiroptical properties and phenomena are embedded in the dynamic polarizabilities (Fig. 3). Specifically, extinction (Abs) and CD are considered, which are calculated as Abs = (σ_+_ + σ_−_)/2 and CD = σ_+_ − σ_−_, where σ_±_ is the extinction cross-section of LCP and RCP incident light.

Chiroptical responses are often classified into intrinsic chirality and extrinsic chirality; the former originates from the geometric chirality of the system, and the latter originates from the illumination conditions. Intrinsic chirality is observed from chiral particles, such as a helical assembly of Au nanospheres (Fig. 3a), which exhibits a nonzero 〈CD〉_Ω_, where 〈 〉_Ω_ is an orientation-averaged quantity. 〈CD〉_Ω_ is directly related to the nonorthogonal ED and MD^31^ and can be determined by the imaginary part of the trace of *α*_c_, Im[Tr(*α*_c_)] = Im[(*α*_c_)_*xx*_ + (*α*_c_)_*yy*_ + (*α*_c_)_*zz*_]/3; analogously, 〈Abs〉_Ω_ is related to Im[Tr(*α*_e_)] in the same way. This property is visually confirmed by the spectra of 〈CD〉_Ω_ and Im[Tr(*α*_c_)] (Fig. 3a), which exhibit a bisignate feature at ~520 nm. Likewise, the spectra of 〈Abs〉_Ω_ and Im[Tr(*α*_e_)] also exhibit a resonance at ~520 nm. Plasmonic nanoparticles generally have highly anisotropic responses, which can be analyzed using the complete set of *α* tensor components.

Although the 〈CD〉_Ω_ of an achiral object is zero, a chiroptical response has been observed when the incident wave vector **k** does not lie on the plane of symmetry. This chiroptical phenomenon, called extrinsic chirality^32,33^, has been studied for a split-ring resonator (SRR), which is apparently achiral due to its mirror symmetry planes (i.e., the *xy*- and *yz*-planes) (Fig. 3b). An SRR is a canonical meta-atom with a magnetoelectric response: an incident electric field polarized in the *x*-direction, *E*_*x*_, generates a current loop, which subsequently generates an MD mode oriented in the *z*-direction *m*_*z*_; this transition corresponds to a nonzero (*α*_c_)_*xz*_^26^. An SRR exhibits an asymmetric response arising from the LCP and RCP light that propagates outside of the mirror planes (Fig. 3b). The CD spectrum peak at 580 nm corresponds to Im[(*α*_c_)_*xz*_], and the Abs peaks at 480 and 580 nm correspond to Im[(*α*_e_)_*xx*_] and Im[(*α*_e_)_*yy*_], respectively. Interestingly, the extrinsic chirality originates from (*α*_c_)_*xz*_, in which the ED mode in the *x*-direction is involved, although (*α*_e_)_*yy*_ is stronger than (*α*_e_)_*xx*_.

The general definition of a chiral object, i.e., one that is not superimposable onto its mirror image using only rotations and translations, has been useful for predicting whether chiral objects have intrinsic chirality. However, it fails to explain the extrinsic chirality of achiral objects for which only orientation-averaged chiroptical responses are observed, such as for randomly dispersed chiral molecules and colloids. As the examples above show, the origins of both intrinsic and extrinsic chirality can be unambiguously explained using the *α*_c_ of subwavelength plasmonic particles. The dipole approximation is the simplest form of the so-called multipole approach, which has been used to extensively explain nanophotonic phenomena, including directional scattering^34^ and Fano resonances^35^, and allows group-theoretical analysis due to the well-defined symmetries of multipoles^36^. However, the dipole approximation must be used with caution. Recently investigated coupled plasmonic clusters^37^, high refractive-index particles^38^, and particles under near-field interactions^39,40^ involve higher-order multipolar transitions, which need to be treated using the higher-order polarizability tensor^27^ or *T*-matrix method^41^. The chiroptical effects of plasmonic particles are sensitive to their geometry^16,42^ and illumination conditions (i.e., highly anisotropic)^43^, so an ensemble-averaged response may strongly differ from a single-particle response^44,45^. Formulations for higher-order multipoles are discussed in ref. ^46^, and anisotropic and higher-order transitional aspects of chiroptical responses are discussed in ref. ^47^.

### Maximally chiral particles

The chiroptical parameters (*α*_c_ and *κ*) provide a method of clearly interpreting chiroptical phenomena. This theoretical framework also allows us to define and study the concept of maximally chiral systems^48^. Under the dipole approximation, maximally chiral particles (MCPs) have polarizabilities that satisfy *α*_e_ = *α*_m_ = ±*α*_c_ and interact with LCP light while being completely transparent to RCP light (or vice versa depending on the sign). In addition, an MCP is dually symmetric; that is, it is excited by and emits light with identical helicity^49,50^. Therefore, an MCP must be completely decoupled from its enantiomer because photons radiated from the former cannot excite the latter. Using these properties, MCPs could be used as helicity filtering media, which scatter light with one helicity while being completely transparent to light with the other helicity. Similar to the dipolar particle case, a formulation that provides a local description of such systems is *ϵ*_r_ = *μ*_r_ = ±*κ*^48^. Such systems have yet to be realized experimentally; however, this is an example of how a strong theoretical framework can guide research towards potential limits and applications.

### Interpretation of plasmonic chiral assemblies: plasmon hybridization theory and coupled-mode theory

In general, assemblies of discrete plasmonic nanoparticles exhibit much stronger chiroptical responses than single continuous plasmonic nanoparticles. Under the multipole framework, this observation can be explained by the increased higher-order multipolar transitions of the coupled plasmonic clusters^37^, but it does not explain the EM interactions between the discrete particles that amplify the chiroptical responses. These interparticle EM interactions have been interpreted using plasmon hybridization theory^51^ and coupled-mode theory^52^.

**Fig. 4:**
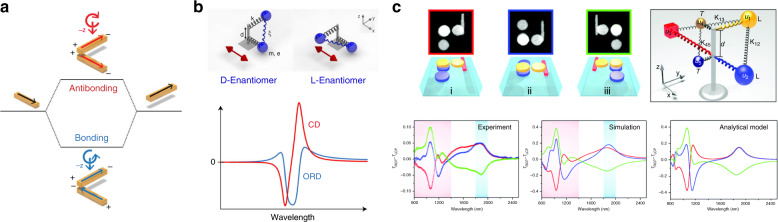
Theoretical frameworks illustrating the strong chiroptical phenomena in subwavelength chiral plasmonic systems. **a** Plasmon hybridization theory and **b** coupled-mode theory, specifically the Born–Kuhn model, describing the electromagnetic chiral response of the coupled nanorod system shown in **a**. **c** Coupled-mode theory describing the chiroptical response of a complicated coupled system. **a**, **b** Reprinted with permission from ref. ^53^, copyright 2013, ACS. **c** Adapted with permission from ref. ^52^, copyright 2015, RSC

Plasmon hybridization theory provides an intuitive picture of the coupled configuration based on the hybridization of isolated plasmonic modes^53,54^. Two twisted plasmonic nanorods (Fig. 4a) exhibit splitting of isolated plasmonic modes into bonding and antibonding states that asymmetrically interact with light with different helicities. This framework provides the charge density distribution for the hybridized modes and produces an acceptable prediction of the coupled state eigenfrequency; however, it is based on an electrostatic approach, so its accuracy decreases as the system size increases.

Coupled-mode theory, also known as the coupled oscillator model, allows for a quantitative analysis of the interparticle EM interactions. The constituent particles are treated as oscillators coupled to each other with coupling strengths acquired through a fitting procedure. Notably, the Born–Kuhn model, which consists of two identical coupled oscillators, has been used to accurately interpret simple chiral systems such as twisted nanorods (Fig. 4b)^53^. This model accurately reconstructs the bisignate chiral signatures and the CD and ORD that are related by the Kramers–Kronig relation. This process can be extended to more complicated systems where many particles are coupled (Fig. 4c), resulting in an excellent quantitative analysis. However, due to the fitting procedure, it is not possible to predict an unknown response from known constituents using this method.

## Chiroptical sensing and plasmon-enhanced circular dichroism

Enantioselective spectroscopy is a potentially useful technique for distinguishing the handedness of chiral molecules in pharmaceutics and synthetic chemistry, but molecular chiroptical signals are intrinsically weak due to the large size mismatch between molecules and the wavelength of light. Research on the ultrasensitive measurement of a small number of chiral biomolecules near plasmonic nanostructures has been undertaken^55–59^. An enhancement factor of 10^6^^60^, a sensitivity of zepto-(10^−21^) M^61,62^, and analysis of the higher-order structure of large biomolecules^63^ have been reported. The experimental aspects of the platforms used to sense chirality have been covered in numerous reviews^10,64^; thus, here, we focus on the theoretical aspects of chiroptical molecular sensing, including the plasmonic antenna effect, local field gradients, superchiral fields, and structural perturbations in plasmonic systems.

### Near-field radiative coupling and antenna effect

Within the semiclassical approach, molecules excited by an off-resonant weak EM field are approximated to a two-level point-like system (i.e., a quantum resonator)^65^. The polarizabilities of chiral molecules are^66^ $\alpha _{\mathrm{e}}\left( \omega \right) = {\it{\epsilon }}^{ - 1}f\left( \omega \right)\left( {{\mathbf{p}}_{12} \otimes {\mathbf{p}}_{21}} \right)$ and $\alpha _{\mathrm{c}}\left( \omega \right) = - i\eta f\left( \omega \right)\left( {{\mathbf{p}}_{12} \otimes {\mathbf{m}}_{21}} \right)$, where **p**_*ij*_ and **m**_*ij*_ are the electric and magnetic transition dipole matrix elements, respectively, *i*, *j* = {1, 2}, 1 and 2 are the initial and final states, and *f*(*ω*) is the molecular dispersion linewidth with information on molecular resonances. Molecular resonances due to electronic modes occur in the UV regime where electronic CD is observed; likewise, vibronic CD is observed in the IR regime due to vibronic modes. The semiclassical theory of the CD effect of a single chiral molecule states that ${\mathrm{CD}}\; \propto \;{\mathrm{Im}}\left( {{\mathbf{p}}_{12} \cdot {\mathbf{m}}_{21}} \right)$, which is related to Im[Tr(*α*_c_)]. This framework within the dipole approximation has also been used to model chiral molecules to study plasmon-enhanced circular dichroism^23^, helical dichroism^67^ and optical forces on chiral molecules^68^, and has been extended to higher-order multipolar transitions that couple to rapidly varying fields^69–71^.

**Fig. 5:**
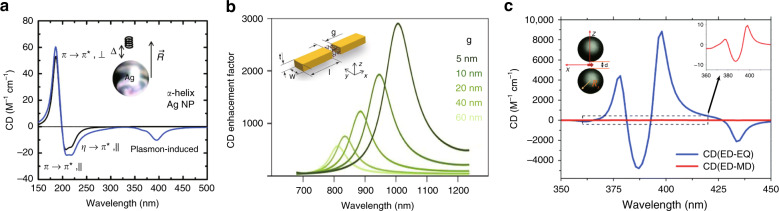
Theoretical reconstruction of PECD. **a** A dipolar chiral molecule near a plasmonic sphere, **b** a chiral medium embedded in a plasmonic gap antenna, and **c** a chiral molecule beyond the dipolar transition embedded in a plasmonic dimer. **a** Adapted with permission from ref. ^23^, copyright 2010, ACS. **b** Adapted with permission from ref. ^72^, copyright 2016, ACS. **c** Adapted with permission from ref. ^70^, copyright 2017, RSC

Chiral molecules adsorbed on plasmonic nanoparticles exhibit strongly amplified chiroptical responses in the visible regime where the localized surface plasmon resonates. This phenomenon has been theoretically reconstructed by placing a chiral molecule near a plasmonic nanoparticle^23,24^ or inside a plasmonic dimer^25^, where the chiral molecules and nanoparticles are radiatively coupled in the near-field. An α-helix near a Ag nanoparticle (Fig. 5a) shows plasmon-enhanced circular dichroism (PECD) near 400 nm, as well as molecular chiroptical signals near 200 nm. This PECD comes from the electrostatic Coulomb interaction between the chiral molecule and the plasmonic nanoparticle. Similarly, the amplified chiroptical responses near plasmonic structures have been reconstructed for chiral molecular media using the finite element method^72–74^ (Fig. 5b). To recognize the microscopic origin of PECD, the observed CD is decomposed into ${\mathrm{CD}} = {\mathrm{CD}}_{{\mathrm{abs}}} + {\mathrm{CD}}_{{\mathrm{sca}}} = {\mathrm{CD}}_{{\mathrm{abs}}}^{{\mathrm{NP}}} + {\mathrm{CD}}_{{\mathrm{abs}}}^{{\mathrm{molecule}}} + {\mathrm{CD}}_{{\mathrm{sca}}}$, where CD_abs_ originates from absorptive losses, CD_sca_ from scattering losses, ${\mathrm{CD}}_{{\mathrm{abs}}}^{{\mathrm{NP}}}$ from absorptive losses of the nanoparticle, and ${\mathrm{CD}}_{{\mathrm{abs}}}^{{\mathrm{molecule}}}$ from absorptive losses of the molecule. Extinction due to weak scatterers (i.e., molecules) occurs dominantly via absorption because their radiative scattering loss is negligible; however, the theoretically observed PECD for a system of chiral molecules and a plasmonic nanoantenna has a strong scattering contribution from the plasmonic nanoantenna^73,74^. These theoretical studies suggest that enantioselective perturbation of the nanoparticle by the chiral molecule could be essential for ultrasensitive chiral sensing, where the nanoparticle acts as a nanoantenna that amplifies the weak molecular chiroptical signals. PECD studies generally considered chiral molecules that are off-resonant with plasmonic nanoparticles, but molecules that are on-resonant with nanostructures have been recently studied using an Al nanoparticle resonant at a UV wavelength^75^ and a cavity resonant at a mid-IR wavelength^76^.

### Local field gradients

Although contributions from the ED–EQ transition to chiroptical responses are comparable to the ED–MD contributions, small chiral scattering systems have generally been modeled based on ED–MD transitions^23–25,67^ because the ED–EQ contribution vanishes due to orientation averaging. Because ED–EQ transitions take local field gradient into account, the ED–EQ contribution to chiroptical responses may not be negligible near plasmonic nanogaps. Plasmonic nanogaps support field enhancement of several orders of magnitude at a singular point, so the fields vary rapidly, and a strong local field gradient is present. This local field gradient was studied for a Ag dimer with an ED–EQ transition^70^ (Fig. 5c), where higher-order transitions could be excited and yield strongly increased chiroptical responses, far exceeding those from ED–MD transitions alone^71^.

### Superchiral fields

Another widely studied mechanism for enantioselective molecular sensing is the superchiral field. The quantification of the chirality of EM fields has been conceptualized only recently as optical chirality^22^. This pseudoscalar field quantity, also called Lipkin’s 00-zilch, was discovered decades ago^77^, but its physical meaning was not fully understood. In a vacuum, optical chirality is locally conserved by the continuity equation:

4 $$\nabla \cdot {\mathbf{\Phi }}_\chi + \partial _{\boldsymbol{t}}\chi = - \left( {{\mathbf{{\cal{J}}}} \cdot \nabla \times {\mathbf{{\cal{E}}}} + {\mathbf{{\cal{E}}}} \cdot \nabla \times {\mathbf{{\cal{J}}}}} \right)/2$$

and the optical chirality density and its flux can be expressed as^22,78,79^:

5 $$\chi = \left( {\epsilon _0{\mathbf{{\cal{E}}}} \cdot \nabla \times {\mathbf{{\cal{E}}}} + \mu _0{\cal{H}} \cdot \nabla \times {\cal{H}}} \right)/2$$

6 $${\mathbf{\Phi }}_\chi = \left[ {{\mathbf{{\cal{E}}}} \times \left( {\nabla \times {\cal{H}}} \right) - {\cal{H}} \times \left( {\nabla \times {\mathbf{{\cal{E}}}}} \right)} \right]/2$$

where ${\mathbf{{\cal{E}}}}\left( {{\mathbf{r}},t} \right)$ and ${\cal{H}}\left( {{\mathbf{r}},t} \right)$ are the electric and magnetic fields in the time domain, respectively. For monochromatic fields, the time-averaged optical chirality density and its flux can be expressed as:

7 $$\left\langle \chi \right\rangle = \omega {\mathrm{/}}\left( {2c^2} \right){\mathrm{Im}}\left( {{\mathbf{E}} \cdot {\mathbf{H}}^ \ast } \right)$$

8 $$\begin{array}{l}\left\langle {{\mathbf{\Phi }}_\chi } \right\rangle = {\mathrm{Re}}\left[ {{\mathbf{E}} \times \left( {\nabla \times {\mathbf{H}}^ \ast } \right) - {\mathbf{H}}^ \ast \times \left( {\nabla \times {\mathbf{E}}} \right)} \right]/4\\ = \left( {\omega {\mathrm{/}}4} \right){\mathrm{Im}}\left( {\epsilon _0{\mathbf{E}}^ \ast \times {\mathbf{E}} + \mu _0{\mathbf{H}}^ \ast \times {\mathbf{H}}} \right)\end{array}$$

where ${\mathbf{E}}({\mathbf{r}},\omega )$ and ${\mathbf{H}}\left( {{\mathbf{r}},\omega } \right)$ are the complex, time-harmonic fields in the frequency domain.

Generalized expressions for the optical chirality dissipation and density in a lossy medium in the frequency domain were recently introduced by comparing these conservation laws with the Poynting theorem (Table 1)^80^. These expressions allow the optical chirality dissipation to be estimated by measuring the optical chirality flux in the far field^81^. Most recently, optical chirality has been generalized to dispersive materials, including dielectric, plasmonic, and negative index media^82^. These expressions will be especially helpful for characterizing the optical chirality density inside or near lossy plasmonic nanostructures. The optical chirality density may allow us to determine and even quantify the chirality of EM fields. Based on these conservation laws, optical chirality has been interpreted as an intrinsic physical quantity that can be transferred (dissipated) to discrete objects^83–85^, just as EM energy can be transferred between objects and EM fields.

**Table 1:** Comparison between electromagnetic field energy and optical chirality for dispersive, lossy media in the frequency domain^80^

	Energy	Optical chirality
Conservation law	$\nabla \cdot {\mathbf{\Phi }}_{\mathrm{u}} + 2i\omega \left( {u_{\mathrm{e}} - u_{\mathrm{m}}} \right) = - {\mathbf{J}}^ \ast \cdot {\mathbf{E}}$	$\nabla \cdot {\mathbf{\Phi }}_\chi + 2i\omega \left( {\chi _{\mathrm{e}} - \chi _{\mathrm{m}}} \right) = - \left( {{\mathbf{J}}^ \ast \cdot \nabla \times {\mathbf{E}} + {\mathbf{E}} \cdot \nabla \times {\mathbf{J}}^ \ast } \right)/4$
Dissipation	$\nabla \cdot \left\langle {{\mathbf{\Phi }}_{\mathrm{u}}} \right\rangle = 2\omega {\mathrm{Im}}\left( {u_{\mathrm{e}} - u_{\mathrm{m}}} \right) = - \left( {\omega /2} \right)\left[ {{\mathrm{Im}}\left( {\it{\epsilon }} \right)\left| {\mathbf{E}} \right|^2 + {\mathrm{Im}}\left( \mu \right)\left| {\mathbf{H}} \right|^2} \right]$	$\nabla \cdot \left\langle {{\mathbf{\Phi }}_\chi } \right\rangle = 2\omega {\mathrm{Im}}\left( {\chi _{\mathrm{e}} - \chi _{\mathrm{m}}} \right) = - (\omega /2){\mathrm{Im}}\left( {{\it{\epsilon }}\mu } \right){\mathrm{Im}}\left( {{\mathbf{E}} \cdot {\mathbf{H}}^ \ast } \right)$
Density	$\left\langle u \right\rangle = {\mathrm{Re}}\left( {u_{\mathrm{e}} + u_{\mathrm{m}}} \right) = \left[ {{\mathrm{Re}}\left( {\it{\epsilon }} \right)\left| {\mathbf{E}} \right|^2 + {\mathrm{Re}}\left( \mu \right)\left| {\mathbf{H}} \right|^2} \right]/4$	$\left\langle \chi \right\rangle = {\mathrm{Re}}\left( {\chi _{\mathrm{e}} + \chi _{\mathrm{m}}} \right) = \left( {\omega /2} \right){\mathrm{Re}}\left( {\it{\epsilon }} \right){\mathrm{Re}}\left( \mu \right){\mathrm{Im}}\left( {{\mathbf{E}} \cdot {\mathbf{H}}^ \ast } \right)$

Electric field energy density $u_{\mathrm{e}} = ({\mathbf{D}}^ \ast \cdot {\mathbf{E}})/4$, magnetic field energy density $u_{\mathrm{m}} = ({\mathbf{B}} \cdot {\mathbf{H}}^ \ast )/4$, electric optical chirality density $\chi _{\mathrm{e}} = \left( {{\mathbf{D}}^ \ast \cdot \nabla \times {\mathbf{E}} + {\mathbf{E}} \cdot \nabla \times {\mathbf{D}}^ \ast } \right)/8$, magnetic optical chirality density $\chi _{\mathrm{m}} = \left( {{\mathbf{B}} \cdot \nabla \times {\mathbf{H}}^ \ast + {\mathbf{H}}^ \ast \cdot \nabla \times {\mathbf{B}}} \right)/8$, electromagnetic field energy flux (Poynting vector) ${\mathbf{\Phi }}_{\mathrm{u}} = ({\mathbf{E}} \times {\mathbf{H}}^ \ast )/2$, and optical chirality flux ${\mathbf{\Phi }}_\chi = \left[ {{\mathbf{E}} \times \left( {\nabla \times {\mathbf{H}}^ \ast } \right) - {\mathbf{H}}^ \ast \times (\nabla \times {\mathbf{E}})} \right]/4$

The optical chirality density appears in the expressions for enantioselective absorption. Under the dipole approximation, the time-averaged extinction rates of an isotropic chiral molecule under LCP (+) and RCP (−) illumination are^22^:

9 $$\begin{array}{l}\left\langle A \right\rangle ^ \pm = \left( {\omega /2} \right){\mathrm{Im}}\left( {{\mathbf{E}}^ \ast \cdot {\mathbf{p}} + {\mathbf{B}}^ \ast \cdot {\mathbf{m}}} \right)\\ = \left( {\omega {\it{\epsilon }}_0/2} \right){\mathrm{Im}}\left( {\alpha _{\mathrm{e}}} \right)\left| {\mathbf{E}} \right|^2 + \left( {\omega \mu _0/2} \right){\mathrm{Im}}\left( {\alpha _{\mathrm{m}}} \right)\left| {\mathbf{H}} \right|^2 \pm \left( {\omega /c} \right){\mathrm{Im}}\left( {\alpha _{\mathrm{c}}} \right){\mathrm{Im}}\left( {{\mathbf{E}} \cdot {\mathbf{H}}^ \ast } \right)\end{array}$$

and the dissymmetry in the extinction rates is proportional to the optical chirality density as $A^ + - A^ - \propto {\mathrm{Im}}\left( {\alpha _{\mathrm{c}}} \right)\chi$. A similar expression can be found for the local absorption in a chiral medium^71,73,74^:

10 $$\begin{array}{l}\left\langle Q \right\rangle ^ \pm = \left( {\omega /2} \right){\mathrm{Im}}\left( {{\mathbf{E}}^ \ast \cdot {\mathbf{D}} + {\mathbf{H}}^ \ast \cdot {\mathbf{B}}} \right)\\ = \left( {\omega /2} \right)\left[ {{\mathrm{Im}}\left( {\it{\epsilon }} \right)\left| {\mathbf{E}} \right|^2 + {\mathrm{Im}}\left( \mu \right)\left| {\mathbf{H}} \right|^2} \right] \pm \left( {\omega /c} \right){\mathrm{Im}}\left( \kappa \right){\mathrm{Im}}\left( {{\mathbf{E}} \cdot {\mathbf{H}}^ \ast } \right)\end{array}$$

**Fig. 6:**
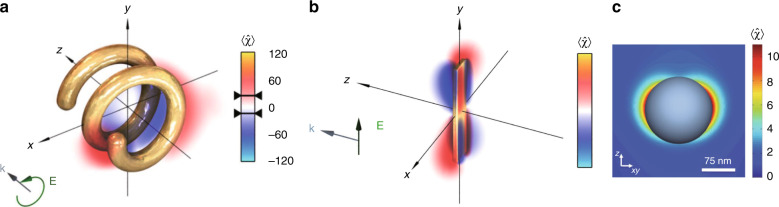
Optical chirality enhancement near nanostructures. **a** Chiral plasmonic, **b** achiral plasmonic, and **c** high refractive-index dielectric particles. **a** Adapted from ref. ^86^, copyright 2012, APS, under the Creative Commons Attribution 3.0 License. **b** Adapted with permission from ref. ^91^ copyright 2012, OSA. **c** Adapted with permission from ref. ^95^, copyright 2013, APS

The optical chirality density in the far field is limited to that of the circularly polarized light but can be enhanced near nanostructures. This so-called superchiral field was suggested as a possible mechanism for the ultrasensitive detection of chiral molecules near nanostructures. High local optical chirality density has been observed near chiral nanostructures^86–89^ (Fig. 6a). However, the chiroptical signals from the chiral nanostructures overwhelm the molecular signals, so the structure background must be compensated for using two opposite^60,62^ or complementary chiral nanostructures^90^. Note that achiral plasmonic nanostructures also support enhanced local optical chirality in the near-field^91,92^ (Fig. 6b). Plasmonic nanostructures enhance the electric field much more strongly than the optical chirality^93^, so the achiral part of the absorption (the first term of Eq. 9) increases more significantly than the chiral part (the last term of Eq. 9). The dissymmetry factor, defined as the ratio between the chiral and achiral components of the absorption, is therefore lowered. Therefore, superchiral light may also refer to fields with a large local field dissymmetry, defined as the local optical chirality divided by the local electric field intensity^22^.

The volume-averaged optical chirality 〈*χ*〉_*v*_ cannot be high near small plasmonic nanoparticles due to their quasi-static nature^94^; 〈*χ*〉_*v*_ may become strong near large chiral structures^86^ but is generally limited due to spatially flipping signs of the local 〈*χ*〉. Strong 〈*χ*〉_*v*_ has been theoretically predicted near high refractive-index dielectric nanostructures with simultaneous electric and magnetic resonances^95–101^ (Fig. 6c). These dielectric nanostructures exhibit strong optical chirality with a weaker electric field enhancement than plasmonic nanostructures. However, the enhancement of the molecular CD from dielectric nanostructures is, to the best of our knowledge, yet to be observed experimentally.

In addition to these limitations, the optical chirality enhancement only explains the enhancement factor of ${\mathrm{CD}}_{{\mathrm{abs}}}^{{\mathrm{molecule}}}$, which is only a small portion of the total CD enhancement factor for a chiral molecule described under the dipole approximation. The local field gradient may explain why ultrasensitive chiral molecular sensing has been demonstrated using plasmonic structures, despite the optical chirality enhancement being limited to 10^2^ and high refractive-index dielectric structures exhibiting strong 〈*χ*〉_*v*_^71^.

Although, based on its conservation laws, optical chirality has been suggested as a measure of field chirality, whether it represents a physical property of an EM field is still controversial^102^. Optical helicity is another potential field quantity that could be used to describe the chirality of EM fields. The optical helicity operator is defined as the spin operator projected onto the linear momentum operator^103^, and optical helicity is interpreted as the number difference between the 1 and −1 spin-polarized photons^102^. In a vacuum, optical helicity is locally conserved as $\nabla \cdot {\mathbf{\Phi }}_h + \partial _th = 0$, where $h = \left( {\eta _0^{ - 1}{\mathbf{A}} \cdot {\mathbf{B}} - \eta _0{\mathbf{C}} \cdot {\mathbf{D}}} \right)/2$ is the optical helicity density and ${\mathbf{\Phi }}_h = \left( {\eta _0^{ - 1}{\mathbf{E}} \times {\mathbf{A}} + \eta _0{\mathbf{H}} \times {\mathbf{C}}} \right)/2$ is its flux, expressed by the electric and magnetic vector potentials^104^, with ${\mathbf{D}} = - \nabla \times {\mathbf{C}} = - {\it{\epsilon }}_0\partial _t{\mathbf{A}}$ and ${\mathbf{B}} = \nabla \times {\mathbf{A}} = - \mu _0\partial _t{\mathbf{C}}$. In a vacuum, ${\mathbf{\Phi}}_{h}$ coincides with the spin angular momentum density flux^102^, and for monochromatic fields, the time-averaged expressions are $\left\langle h \right\rangle = {\mathrm{Im}}\left( {{\mathbf{E}} \cdot {\mathbf{H}}^ \ast } \right)/\left( {2c\omega } \right)$ and $\left\langle {{\mathbf{\Phi }}_h} \right\rangle = c/\left( {4\omega } \right){\mathrm{Im}}\left( {{\it{\epsilon }}_0{\mathbf{E}}^ \ast \times {\mathbf{E}} + \mu _0{\mathbf{H}}^ \ast \times {\mathbf{H}}} \right)$. For monochromatic fields, ${h}$ is proportional to χ, but they are different distinguishable quantities in general^102,105^. ${{h}}$ has been generalized to lossless dispersive media, where dispersion-modified quantities are considered. In such media, ${\mathbf{\Phi}}_h$ and the spin angular momentum density flux, which are proportional to each other in nondispersive media, become completely unrelated^105^. A recent paper^106^ reviewed optical chirality and optical helicity in detail.

### Structural perturbations

**Fig. 7:**
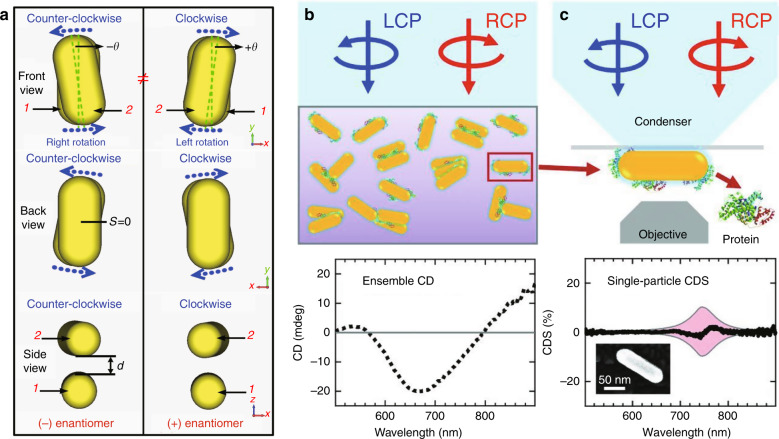
Plasmonic chirality due to structural perturbations. **a** Different handedness of a plasmonic nanorod dimer depending on the handedness of the chiral molecules. Measurements of **b** ensemble and **c** single-particle chiroptical signals. **a** Adapted with permission from ref. ^19^, copyright 2013, NPG. **b**, **c** Reprinted with permission from ref. ^107^, copyright 2019, AAAS

Structural perturbations in plasmonic systems arising from chiral molecules have led to strong chiroptical responses originating from the induced structural chirality^59^, where the resulting handedness of the dimerized plasmonic nanorods depends on the handedness of the molecules (Fig. 7a). Theoretical investigations of this phenomenon did not directly include the chiroptical properties of the chiral molecules because the chiroptical signals from the plasmonic systems far exceed the molecular signals. Because the shapes of the dimerized nanorods are not identical, the responses from an individual particle and an ensemble of particles are different. Recently, this was investigated at the single-particle level, where an ensemble of nanorods (Fig. 7b) exhibited a stronger, broader chiroptical response than that of a single nanorod when coupled to chiral molecules (Fig. 7c)^107^. The statistical nature of this phenomenon may dominate when plasmonic systems are structurally unstable.

Recently, enantioselective molecular sensing assisted by Ag chiral nanoparticles (AgCNPs) was also demonstrated in the deep-UV region^108^, where the effects of superchiral fields are absent. A helical arrangement of core/surface AgCNPs cannot generate a localized surface plasmon resonance (LSPR) at this wavelength range, even though they exhibit plasmonic chirality in the visible spectrum. Here, the enantioselective CD amplification is attributed to the enantiospecific change in the dihedral angle of the binaphthyl chromophore when adsorbed on the AgCNPs through the Ag-S bicontacts. Similarly, this kind of chirality transfer of the helical topography from the AgCNPs to the adsorbed molecules also enables the formation of supramolecular chiral complexes such that the CD signal of the molecules is increased^109^.

## Chiroptical manipulation

This section discusses the momentum exchange in light–matter interactions that produces optical forces and torques. First, we introduce the Maxwell stress tensor, which enables the calculation of optical forces and torques. Then, we derive the formulas of the optical force and torque exerted on a chiral sphere, examine how the optical force and torque affect a chiral object and review experimental demonstrations of light-driven particle control.

### Optical forces and torques

Light carries linear and angular momenta that can be exchanged with objects. Therefore, the use of light to capture, trap, pull, push, rotate, and guide microscopic objects has been explored^110–121^. It is a challenge to produce an analytic formula for complex systems that describes the optical forces and torques exerted on an object in an EM field, but fortunately, they can be calculated in a systematic way using stress tensors^21,122–124^. The most commonly used is the Maxwell stress tensor, also known as the Minkowski stress tensor^124–126^. In a homogeneous medium, it is defined as:

11 $$\overleftrightarrow{\mathbf{T}}\left( {{\mathbf{r}},{t}} \right) = {\mathbf{{\cal{D}}}}{\cal{E}} + {\mathbf{{\cal{B}}}}{\cal{H}} - \frac{1}{2}\overleftrightarrow{\mathbf{I}}\left( {{\mathbf{{\cal{D}}}} \cdot {\cal{E}} + {\mathbf{{\cal{B}}}} \cdot {\cal{H}}} \right)$$

where ${\mathbf{{\cal{D}}}}\left( {{\mathbf{r}},t} \right)$ and ${\mathbf{{\cal{B}}}}({\mathbf{r}},t)$ are the electric displacement field and the magnetic induction field in the time domain, respectively, and $\overleftrightarrow{\mathbf{I}}$ is a unit tensor. In a region *V*, the mechanical contribution to the force can then be expressed as:

12 $${\mathbf{F}}_{\mathrm{mech}} = \frac{{{\rm{d}}{\mathbf{P}}_{\mathrm{mech}}}}{{{\mathrm{d}}}{t}} = - \frac{1}{{{\it{c}}^2}}{\int\nolimits_{\it{V}}} {\frac{{\partial {\mathbf{S}}}}{{\partial {\it{t}}}}{{\mathrm{d}}}{V} + {\int\nolimits_{V}} {\nabla \cdot \overleftrightarrow{\mathbf{T}}{{\mathrm{d}}}{V}}}$$

where d*V* is an infinitesimal volume of *V*. The first term on the right-hand side is associated with the time derivative of the EM linear momentum and corresponds to an EM force term. Using the divergence theorem, the optical force can be obtained as:

13 $${\mathbf{F}}\left( {{\mathbf{r}},{\it{t}}} \right) = \frac{{{\rm{d}}\left( {{\mathbf{P}}_{\mathrm{mech}} + {\mathbf{P}}_{\mathrm{em}}} \right)}}{{{\mathrm{d}}}{t}} = {\oint_{S}} {\overleftrightarrow{\mathbf{T}} \cdot {\rm{d}}{\mathbf{a}}}$$

where d***a*** is an infinitesimal surface of *V*. The time-averaged force $\left\langle {\mathbf{F}} \right\rangle = {\oint}_S {\left\langle \overleftrightarrow{\mathbf{T}} \right\rangle \cdot {\mathrm{d}}{\boldsymbol{a}}}$ can be calculated using a surface integral over the time-averaged Maxwell stress tensor. Similarly, the torque can also be obtained by using the Maxwell stress tensor:

14 $${\boldsymbol{\Gamma }}_{{\mathrm{mech}}} = - \frac{\partial }{{\partial {\it{t}}}}{\int_{\it{V}}} {{\mathbf{r}} \times \left( {{\mathbf{{\cal{D}}}} \times {\mathbf{{\cal{B}}}}} \right){{\mathrm{d}}}{V} - {\oint_{\it{S}}} {{\mathbf{n}} \cdot \left( {\overleftrightarrow{\mathbf{T}} \times {\mathbf{r}}} \right){\rm{d}}{\mathbf{a}}} }$$

If we consider a steady state such as laser illumination, the first term on the right-hand side, which is a time-derivative term, becomes zero. Therefore, the time-averaged optical torque can be expressed as $\left\langle {{\boldsymbol{\Gamma }}_{{\mathrm{mech}}}} \right\rangle = - {\oint}_S {{\mathbf{n}} \cdot \left\langle {\overleftrightarrow{\mathbf{T}} \times {\mathbf{r}}} \right\rangle {\mathrm{d}}{\mathbf{a}}}$.

### Enantioselective optical forces and torques

Circularly polarized light has been utilized as a tool to mechanically separate chiral objects with opposite handedness. Depending on the helicity of the incident light, it is scattered differently by the object. We examine the time-averaged optical force acting on an object induced by an arbitrary incident wave. For further simplicity, we consider the object to be isotropic and in the regime of the linear, harmonic and dipolar approximation^127–129^. More detailed expressions of the optical force and torque can be found in other studies^130,131^. The time-averaged optical force^21,129^ exerted on a sphere can be expressed as:

15 $$\left\langle {\mathbf{F}} \right\rangle = \frac{1}{2}{\mathrm{Re}}\left[ {\nabla {\mathbf{E}}^ \ast \cdot {\mathbf{p}} + \nabla {\mathbf{B}}^ \ast \cdot {\mathbf{m}} - \frac{{{k}_0^4}}{{6\pi \epsilon _0{\it{c}}}}\left( {{\mathbf{p}} \times {\mathbf{m}}^ \ast } \right)} \right]$$

where $k_0$ is the wavenumber in a vacuum. Substitution of Eq. 2 into Eq. 15 gives the following equations:

16 $$\begin{array}{l}\left\langle {\mathbf{F}} \right\rangle = - \nabla \left\langle {\mathbf{U}} \right\rangle + \frac{\sigma }{c}\left\langle {{\mathbf{\Phi }}_{\mathrm{u}}} \right\rangle + c\nabla \times \left[ {\sigma _{\mathrm{e}}\left\langle {{\mathbf{L}}_{\mathrm{e}}} \right\rangle + \sigma _{\mathrm{m}}\left\langle {{\mathbf{L}}_{\mathrm{m}}} \right\rangle } \right]\\ + \frac{1}{{{c}}}{\rm{Im}} \left( {\alpha _{\mathrm{c}}} \right)\nabla \times \left\langle {{\mathbf{\Phi }}_\textrm{u}} \right\rangle + \frac{{ck_0^5}}{{3\pi }}{\rm{Re}} \left( {\alpha _{\mathrm{e}}\alpha _{\mathrm{c}}^ \ast } \right)\left\langle {{\mathbf{L}}_{\mathrm{e}}} \right\rangle \\ + \frac{{ck_0^5}}{{3\pi }}{\rm{Re}} \left( {\alpha _{\mathrm{m}}\alpha _{\mathrm{c}}^ \ast } \right)\left\langle {{\mathbf{L}}_{\mathrm{m}}} \right\rangle - 4{\mathrm{k}}_0^2{\rm{Im}} (\alpha _{\mathrm{c}})\left\langle {{\mathbf{\Phi }}_h} \right\rangle \end{array}$$

where $\left\langle {\mathbf{U}} \right\rangle = - \frac{{{\it{\epsilon }}_0}}{4}{\mathrm{Re}}\left( {\alpha _{\mathrm{e}}} \right)\left| {\mathbf{E}} \right|^2 - \frac{{\mu _0}}{4}{\mathrm{Re}}\left( {\alpha _{\mathrm{m}}} \right)\left| {\mathbf{H}} \right|^2 + \frac{1}{{2{\mathrm{c}}}}{\mathrm{Re}}\left( {\alpha _{\mathrm{c}}} \right){\mathrm{Im}}\left( {{\mathbf{H}} \cdot {\mathbf{E}}^ \ast } \right)$ is the free energy and $\sigma = \sigma _{\mathrm{e}} + \sigma _{\mathrm{m}} - \frac{{k_0^4}}{{6\pi }}\left[ {{\mathrm{Re}}\left( {\alpha _{\mathrm{e}}\alpha _{\mathrm{m}}^ \ast } \right) - \alpha _{\mathrm{c}}\alpha _{\mathrm{c}}^ \ast } \right],$ where $\sigma _{\mathrm{e}} = k_0{\mathrm{Im}}\left( {\alpha _{\mathrm{e}}} \right)$ and $\sigma _{\mathrm{m}} = k_0{\mathrm{Im}}\left( {\alpha _{\mathrm{m}}} \right)$ are the extinction cross-sections for the electric dipole and magnetic dipole contributions, $\left\langle {{\mathbf{L}}_{\mathrm{e}}} \right\rangle = \frac{{{\it{\epsilon }}_0}}{{4\omega }}{\mathrm{Im}}\left( {{\mathbf{E}} \times {\mathbf{E}}^ \ast } \right)$ and $\left\langle {{\mathbf{L}}_{\mathrm{m}}} \right\rangle = \frac{{\mu _0}}{{4\omega }}{\mathrm{Im}}\left( {{\mathbf{H}} \times {\mathbf{H}}^ \ast } \right)$ are the time-averaged spin-density fluxes, which manifest the polarization states of light, for example, $\left\langle {{\mathbf{L}}_{\mathrm{e}}} \right\rangle = \frac{{{\it{\epsilon }}_0}}{{4\omega }}\left| {E_0} \right|^2$ for LCP light, $\left\langle {{\mathbf{L}}_{\mathrm{e}}} \right\rangle = - \frac{{{\it{\epsilon }}_0}}{{4\omega }}\left| {E_0} \right|^2$ for RCP light and $\left\langle {{\mathbf{L}}_{\mathrm{e}}} \right\rangle = 0$ for linearly polarized light. The summation of the two time-averaged spin-density fluxes is directly associated with the time-averaged optical helicity flux in a vacuum: $\left\langle {{\mathbf{L}}_{\mathrm{e}}} \right\rangle + \left\langle {{\mathbf{L}}_{\mathrm{m}}} \right\rangle = \left\langle {{\mathbf{\Phi }}_h} \right\rangle /c$. In Eq. 16, $- \nabla \left\langle {\mathbf{U}} \right\rangle$ represents the gradient force, $\frac{\sigma }{c}\left\langle {{\mathrm{\Phi }}_{\mathrm{u}}} \right\rangle$ represents the radiation pressure, $c\nabla \times \left[ {\sigma _{\mathrm{e}}\left\langle {{\mathbf{L}}_{\mathrm{e}}} \right\rangle + \sigma _{\mathrm{m}}\left\langle {{\mathbf{L}}_{\mathrm{m}}} \right\rangle } \right]$ represents curl-spin forces, which are related to the curl of the spin-density fluxes, and $\frac{1}{{{c}}}{\mathrm{Im}}\left( {\alpha _{\mathrm{c}}} \right)\nabla \times \left\langle {{\mathbf{\Phi }}_\textrm{u}} \right\rangle$ corresponds to a vortex force that originates from the energy flow vortex. The last two terms are spin-density forces, which are related to the coupling of the chiral object and spin-density fluxes.

Two factors contribute to the helicity dependent force. The first originates from the polarization states of light. If circularly polarized light illuminates an achiral object, then the nonzero spin-density fluxes give rise to a curl-spin force and a spin-density force in a direction that depends on the spin-density fluxes. The second contribution results from the *α*_c_ of an object. Under the same illumination, a chiral object and its enantiomer experience different vortex and spin-density forces, as implied by the *α*_c_ term in Eq. 16.

Light also carries angular momentum and therefore can apply a torque. The time-averaged optical torque exerted on an arbitrary object can be expressed as^21,128^:

17 $$\left\langle {\mathbf{\Gamma }} \right\rangle = \frac{1}{2}{\mathrm{Re}}\left( {{\mathbf{p}} \times {\mathbf{E}}^ \ast + {\mathbf{m}} \times {\mathbf{B}}^ \ast } \right) + \frac{{{\it{k}}_0^3}}{{12\pi }}\left[ {\frac{1}{{\epsilon _0}}{\mathrm{Im}}\left( {{\mathbf{p}}^ \ast \times {\mathbf{p}}} \right) + \mu _0{\mathrm{Im}}\left( {{\mathbf{m}}^ \ast \times {\mathbf{m}}} \right)} \right]$$

Then, the optical torque can be expressed in terms of the polarizabilities and EM fields by substituting Eq. 2 into Eq. 17 to yield:

18 $$\begin{array}{l}\left\langle {\mathbf{\Gamma }} \right\rangle = \left[ {\frac{2}{c}{\rm{Im}}\left( {\alpha _{\rm{c}}} \right) - \frac{{{{k}}_0^3}}{{3{\it{\uppi }}{\mathrm{c}}}}{\rm{Re}} \left( {\alpha _{\mathrm{e}}\alpha _{\mathrm{c}}^ \ast } \right) - \frac{{k_0^3}}{{3\pi c}}{\rm{Re}} \left( {\alpha _{\mathrm{m}}\alpha _{\mathrm{c}}^{\ast}} \right)} \right]\left\langle {\mathbf{\Phi}}_{\rm{u}} \right\rangle \\ + \left[ {\frac{{k_0^3}}{{6\pi c}}{\rm{Im}} \left( {\alpha _{\mathrm{e}}\alpha _{\mathrm{c}}^{\ast}} \right) - \frac{{k_0^3}}{{6\pi c}}{\rm{Im}} \left( {\alpha _{\mathrm{m}}\alpha _{\mathrm{c}}^{\ast}} \right)} \right]{\rm{Im}} \left( {\mathbf{E} \times {\mathbf{H}}^{\ast}} \right)\\ + \left[ {\frac{{\omega k_0^3}}{{3\pi }}\alpha _{\mathrm{e}}\alpha _{\mathrm{e}}^{\ast} + \frac{{\omega k_0^3}}{{3\pi }}\alpha _{\mathrm{c}}\alpha _{\mathrm{c}}^{\ast} - 2\omega {\rm{Im}} (\alpha _{\mathrm{e}})} \right]\left\langle {{\mathbf{L}}_{\mathrm{e}}} \right\rangle \\ + \left[ {\frac{{\omega k_0^3}}{{3\pi}}\alpha _{\mathrm{m}}\alpha _{\mathrm{m}}^{\ast} + \frac{{\omega k_0^3}}{{3\pi }}\alpha _{\mathrm{c}}\alpha _{\mathrm{c}}^{\ast} - 2\omega {\rm{Im}} (\alpha _{\mathrm{m}})} \right]\left\langle {{\mathbf{L}}_{\mathrm{m}}} \right\rangle \end{array}$$

The first term is the radiation torque, which is exclusively determined by the coupling of the chiral object and the Poynting vector. The second term is associated with the alternating flow of the stored energy and is zero for a plane wave due to the term ${\mathrm{Im}}\left( {{\mathbf{E}} \times {\mathbf{H}}^ \ast } \right)$. The last two terms correspond to the electric and magnetic contributions to the spin torque arising from the spin-density fluxes, respectively.

Similarly, the helicity dependent optical torque is attributed to two factors, the polarization of light and the *a*_c_ of an object. Therefore, enantioselective control of an object with light is possible in three distinct instances: (1) achiral light with a chiral object, (2) chiral light with an achiral object, and (3) chiral light with a chiral object. In the following section, we review numerical investigations and experimental demonstrations of these three cases.

### Experimental demonstrations of enantioselective separation

**Fig. 8:**
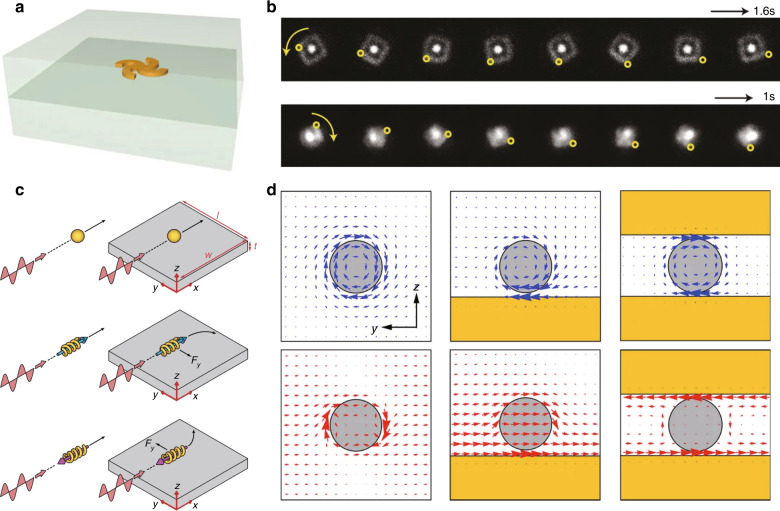
Optical force and torque. **a** Illustration and **b** dark-field microscopy image under 810nm (top) and 1700 nm (bottom) illumination of a plasmonic motor^118^. **c** Schematics of optical forces applied to an achiral (top), right-handed (middle), and left-handed (bottom) object placed on a substrate, and **d** corresponding time-averaged Poynting vector (top) and time-averaged electric spin density **L**_e_ (bottom)^133^. **a**, **b** Reprinted with permission from ref. ^118^, copyright 2010, NPG. **c**, **d** Reprinted with permission from ref. ^133^, copyright 2014, NPG, under a Creative Commons license

Achiral linearly polarized light interacts with chiral objects and their enantiomers differently. An interesting example is a light-driven motor^118^. Linearly polarized light can rotate a gammadion-shaped gold structure embedded in a silica block as a motor (Fig. 8a). The planar gammadion structure is achiral^132^, but the broken in-plane mirror symmetry gives rise to a chiral distribution of the Poynting vector and a resultant optical torque. The increased cross-section at the resonant frequencies leads to rotation of the whole microsized sample (Fig. 8b), where the rotation velocity and direction are controlled by tuning the incident wavelength. Interactions between achiral light and chiral objects also give rise to another type of enantioselective optical force. A linearly polarized plane wave in an isotropic medium has constant field amplitude and time-averaged Poynting vector. Closer examination of the optical forces (Eq. 16) shows that no enantioselective optical force exists. A chiral object would only experience a longitudinal force associated with the radiation pressure^133^. However, a chiral object placed on a substrate experiences an additional lateral optical force (Fig. 8c). The reflected fields break the symmetry of the Poynting vector (Fig. 8d), which leads to a lateral radiation pressure. Achiral light has also been used to detect enantiomers of naturally occurring chiral materials, for example, gas molecules using linearly polarized light^134,135^, and carbon nanotubes using unpolarized light^135^.

The second instance occurs when chiral light interacts with an achiral object. An evanescent field can also apply a lateral force on an achiral object^136^. A propagating wave has momentum and spin associated with the wave vector and polarization, respectively. The spin momenta of the propagating waves cancel out due to symmetry. In contrast, a single evanescent wave has a momentum component determined by the circular polarization of the wave. The spin of the evanescent wave is independent of the polarization while it is transverse to the wave vector. Vertical inhomogeneity of the evanescent fields makes the spin momentum depend on the transverse helicity. This inhibits the cancellation of the spin momenta and thus produces a helicity dependent lateral force and torque.

Optical forces and torques also occur when the light and object are both chiral. In such cases, enantioselective separation occurs as a result of the spin-density fluxes of the light and the *a*_c_ of the object. Helicity dependent sorting of microsized objects with broken mirror symmetry was experimentally demonstrated in a fluidic environment^137^. Another lateral force mechanism is associated with a direct interaction between circular polarization and a chiral object^138^. The direction of the lateral force is perpendicular to the propagation direction of evanescent waves. A chiral object in an evanescent field experiences a lateral force, where the direction depends on the handedness. These helicity dependent lateral forces suggest the possibility of enantiomer-selective sorting.

Circularly polarized light can also trap a chiral object enantioselectively. Three-dimensional trapping of a chiral liquid crystal droplet under two counterpropagating circularly polarized beams has been verified in both numerical simulations and experiments^139^. The trapping depends on the helicity of the incident fields. Droplets are captured when they have the same handedness as the light, but droplets of a certain size are trapped if their helicity is opposite to that of the light.

The enantioselective optical force and torque facilitate light-driven capturing, pushing, pulling, guiding and even sorting of chiral objects, which is extremely challenging to achieve by other means. These chiroptical manipulations can be applied to control targets from microsized artificial objects to molecules and solid, liquid or gaseous objects. The chiroptical force and torque are weak and thus only affect microscale targets. To be applicable to practical applications involving the control of larger and heavier objects, the chiroptical force and torque could be increased using surface plasmon polaritons. This improvement would provide an effective way to control larger chiral objects in a variety of applications in physics, chemistry, and biology.

## Interactions between chiral matter and helical electromagnetic fields

### Orbital angular momentum of light

Another property that gives chiral properties to light is orbital angular momentum (OAM)^140^. This extra degree of freedom enriches light–matter interactions or light-light interactions and generates many intriguing phenomena, such as spin-orbit interactions or helical dichroism. In this section, we discuss how OAM is related to circular polarization and how it interacts with chiral objects.

The linear momentum of light is defined as^126^:

19 $${\mathbf{P}} = \epsilon _0{\int} {{\mathbf{E}} \times {\mathbf{B}}\;{\mathrm{d}}V} $$

This formula naturally gives the angular momentum of light **J**:

20 $$\begin{array}{l}{\mathbf{J}} =\displaystyle \epsilon _0{\int} {{\mathbf{r}} \times \left( {{\mathbf{E}} \times {\mathbf{B}}} \right){\mathrm{d}}V} \\ = \displaystyle \epsilon _0{\int} {\mathop {\sum}\limits_i {E_i\left( {r \times \nabla } \right)A_i{\mathrm{d}}V + \epsilon _0{\int} {{\mathbf{E}} \times {\mathbf{A}}\;{dV}}}} \\ = {\mathbf{L}} + {\mathbf{S}}\end{array}$$

**J** can be separated into two parts: orbital (**L**) and spin (**S**), often called OAM and spin angular momentum (SAM), respectively^141,142^. Since the choice of gauge can change OAM and SAM while not affecting their sum, the validity of the separation has been debated^143–147^. Equation 20 shows the gauge-independent form of OAM and SAM, and both momenta are no longer regarded as physical and measurable quantities.

### Formulation of helical fields

**Fig. 9:**
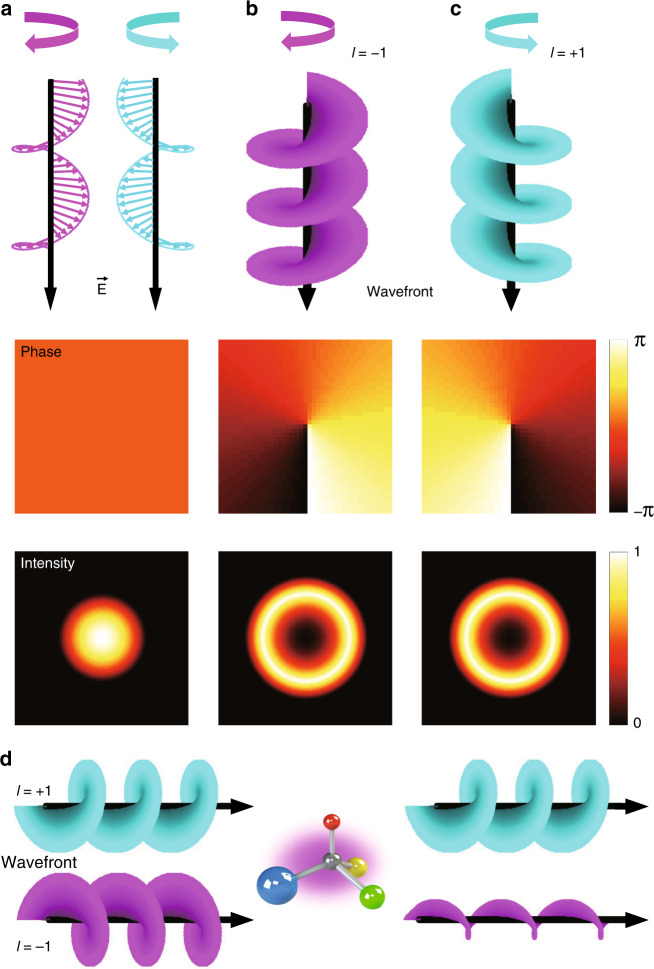
Illustrations of chiral light. **a** Circularly polarized light with *l*=0. Top row: spatial distribution of electric fields, middle row: cross-section of the phase distribution, bottom row: cross-section of the intensity distribution. Light carrying **b** *l*=−1 and **c** *l*=+1 OAM. Top row: wavefront, middle row: cross-section of the phase distribution, and bottom row: cross-section of the intensity distribution. **d** Illustrations of helical dichroism. Light carrying *l*=+1 (blue) OAM interacts weakly with the chiral object, while light with *l*=−1 (pink) interacts strongly and is absorbed

To see how the angular momenta are related to the propagation properties of light, we express the field profile of light propagating along the *z*-axis in cylindrical coordinates within the paraxial approximation as^148^:

21 $${\mathbf{E}}_{kl}\left( {r,\phi ,z,t} \right) = {\hat{\mathbf e}}u\left( r \right)e^{i(kz - \omega t)}e^{ - il\phi }$$

where *u*(*r*) is a scalar function that determines the radial distribution, and *l* is an integer often called the “topological charge” and is associated with OAM. The z-component of the OAM operator can be defined as $\hat L_z = - i\frac{\partial }{{\partial \phi }}$, analogous to quantum mechanics. The eigenmodes of $\hat L_z$ are vortices with eigenvalue $L_z = l\hbar$. Thus, *L*_*z*_ represents the OAM that the light carries. The radial scalar function is mostly described in terms of the Laguerre–Gaussian mode^149^ and Bessel modes^150,151^, both of which converge to the same value at the beam axis. ${\hat{\mathbf e}}$ is a polarization vector associated with SAM. For circularly polarized light, the polarization vector is $${\hat{\mathbf e}} = \frac{1}{{\sqrt 2 }}( {\begin{array}{*{20}{c}} 1 \\ {i\sigma } \end{array}})$$, where *σ* = 1 for LCP light and *σ* = −1 for RCP light. Circularly polarized light carries $\hat S_z = \sigma \hbar$ of SAM per photon, where *S*_*z*_ is the eigenvalue of the *z*-component of the SAM operator $\hat S_z$ and is directly related to the polarization state. Light that carries a nonzero σ is chiral in the sense that the tip of the electric field rotates either clockwise or counterclockwise in space and time as the wave propagates, which, when drawn, results in enantiomeric spirals. If *l* = 0, then the cross-sectional phase distribution of the fields is constant because of the plane wavefront, and the intensity distribution has a circular shape (Fig. 9a). In contrast, if *l* ≠ 0, then the wavefront is helical and is often called ‘twisted light’. The wavefront and cross-sectional phase and intensity profiles when *l* = −1 and *l* = +1 are plotted in Fig. 9b, c. The spatially varying phase profile along the transverse plane originates from the azimuthal phase profile term $e^{ - il\phi }$, and the intensity profile is toroidal as a result of the singularity on the beam axis.

Neither OAM nor SAM are a measure of the total angular momentum, as their names may imply. Instead, the sum of OAM and SAM is the total angular momentum of light^141,142,152^ and becomes a generator of a simple rotation. The inherent link between the two angular momenta, often called the spin-orbit interaction, produces many interesting chiral phenomena that involve OAM. Without even considering the total angular momentum, we can imagine that twisted light interacts differently with chiral objects depending on *l*, as circularly polarized light does, because the helical wavefront of twisted light lacks mirror symmetry (Fig. 9b, c, top row).

Interactions between SAM and chiral objects can be proven within the dipole description. However, the subject of whether light with OAM interacts with chiral objects within the dipole approximation has been controversial. Considering only electric and magnetic dipoles, some theoretical papers have predicted coupling between OAM and chirality^150,153,154^, whereas others have reached the opposite conclusion where the dipolar contributions cancel^148,155–157^. The latter result has been verified experimentally^158,159^. However, by taking higher-order terms such as the electric quadrupole moments into account, the existence of an interaction between OAM and chirality has been proven theoretically and is comparable to spin-induced chirality^155,160^.

### Helical dichroism

OAM has been generally excluded when discussing chiral responses such as CD. However, these chiral responses are also subject to OAM. Twisted light incident on a plasmonic helix exhibits strongly enhanced circular dichroism, which is induced by the OAM-chiral interaction^161^. In other words, the absorption difference between light with opposite SAM and the same nonzero OAM can be strongly amplified or shifted. As in circular dichroism, which is a manifestation of the interaction between circularly polarized light and a chiral object, oppositely twisted light interacting with a chiral object may show an absorption difference. Recently, helical dichroism or OAM dichroism, as an analog of circular dichroism, has been defined as the transmission or absorption difference of oppositely twisted beams interacting with chiral objects. The chiral object interacts strongly with one OAM state and barely interacts with the opposite state. The different strengths of these light–matter interactions result in different absorptions, as illustrated in Fig. 9d. Helical dichroism has been theoretically^67,162^ and experimentally^163^ demonstrated. Recently, numerical studies on helical dichroism for single photon absorption revealed that helical dichroism depends on σ*l* and is therefore invariant when both SAM and OAM are flipped^160^: $\left( {\sigma ,l} \right) \to ( - \sigma , - l)$. Furthermore, a chiro-optical response has been observed in the interaction of twisted beams and achiral structured objects^164,165^ and even achiral atomic matter^166^. The possibility of trapping a chiral object using light with OAM has also been suggested^139^. Understanding the OAM transfer from light to chiral objects may deepen our understanding of chiral matter interactions and may enable practical applications, including the sensing of enantiomers and the detection of molecular chirality.

## Conclusions

In this review, we presented theoretical frameworks of EM chiral systems and descriptions of chiroptical phenomena. The EM chirality of microscopic chiral particles and macroscopic chiral media is modeled by the magnetoelectric coupling terms (*α*_c_ and *κ*), and the chirality of light is characterized by the local handedness density (*χ* or ${h}$) and its flux (${\mathbf{\Phi}}_\chi$ or ${\mathbf{\Phi}}_{h}$). Several different chiroptical phenomena were discussed in terms of the chiroptical parameters of light and matter defined above. We further discussed light carrying OAM, chiroptical forces and torques, and OAM-dependent scattering.

In general, studies on chiroptical effects only consider geometrically chiral systems (intrinsic chirality), but chiroptical effects are also present in achiral systems (extrinsic chirality). Both intrinsic and extrinsic chirality can be interpreted using magnetoelectric terms such as *α*_c_. In addition, globally achiral light can be locally chiral near nanostructures or surfaces, so chiral objects undergo different light–matter interactions. This participation of achiral systems and fields in enantioselective light–matter interactions indicates that conventional concepts of chiroptical systems need to be extended. Firm theoretical backgrounds are necessary for interpreting the mechanisms behind complicated chiroptical effects, as illustrated in the discussion about the controversies in plasmon-enhanced chiral sensing processes.

Not only absorptive and scattering phenomena but also optomechanical effects can be explained using this theoretical framework. Other chiroptical effects, such as the photothermal effect^167,168^, magnetic circular dichroism^169^, and nonlinear chirality^170,171^, could also be interpreted based on these same outlines. We expect this review to provide a clear understanding of the underlying theory of chiroptical systems and help guide research on and applications of chiroptical phenomena in a theoretically robust manner. Furthermore, we anticipate that the concept of machine learning will be utilized for the automatic design and optimization of chiral structures with desired optical properties^172–175^. This will provide a new perspective to understand and facilitate chiroptical phenomena and devices, and further promote the applications of chiroptical platforms, especially in the fields of, but not limited to, metamaterials^176,177^, sensing^178^, spintronics^179^, and stereochemistry^180^.

## References

[1] Barron LD (1986). True and false chirality and parity violation. Chem. Phys. Lett..

[2] Caloz C, Sihvola A (2020). Electromagnetic chirality, Part 2: the macroscopic perspective [electromagnetic perspectives]. IEEE Antennas Propag. Mag..

[3] Ma XL (2017). Meta-chirality: fundamentals, construction and applications. Nanomaterials.

[4] Hentschel M (2017). Chiral plasmonics. Sci. Adv..

[5] Kuzyk A (2018). DNA origami route for nanophotonics. ACS Photonics.

[6] Liu N, Liedl T (2018). DNA-assembled advanced plasmonic architectures. Chem. Rev..

[7] Sharma V (2009). Structural origin of circularly polarized iridescence in jeweled beetles. Science.

[8] Cecconello A (2017). Chiroplasmonic DNA-based nanostructures. Nat. Rev. Mater..

[9] Ma W (2017). Chiral inorganic nanostructures. Chem. Rev..

[10] Kong XT (2018). Plasmonic chirality and circular dichroism in bioassembled and nonbiological systems: theoretical background and recent progress. Adv. Mater..

[11] Qiu M (2018). 3D metaphotonic nanostructures with intrinsic chirality. Adv. Funct. Mater..

[12] Collins JT (2017). Chirality and chiroptical effects in metal nanostructures: fundamentals and current trends. Adv. Optical Mater..

[13] Lindell IV (1994). Electromagnetic Waves in Chiral and Bi-Isotropic Media.

[14] Gao WL (2015). Topological photonic phase in chiral hyperbolic metamaterials. Phys. Rev. Lett..

[15] Pendry JB (2004). A chiral route to negative refraction. Science.

[16] Fan ZY, Govorov AO (2012). Chiral nanocrystals: plasmonic spectra and circular dichroism. Nano Lett..

[17] Yan WJ (2012). Self-assembly of chiral nanoparticle pyramids with strong *R*/*S* optical activity. J. Am. Chem. Soc..

[18] Kuzyk A (2012). DNA-based self-assembly of chiral plasmonic nanostructures with tailored optical response. Nature.

[19] Ma W (2013). Chiral plasmonics of self-Assembled nanorod dimers. Sci. Rep..

[20] Canaguier-Durand A, Genet C (2015). Chiral route to pulling optical forces and left-handed optical torques. Phys. Rev. A.

[21] Canaguier-Durand A (2013). Mechanical separation of chiral dipoles by chiral light. N. J. Phys..

[22] Tang YQ, Cohen AE (2010). Optical chirality and its interaction with matter. Phys. Rev. Lett..

[23] Govorov AO (2010). Theory of circular dichroism of nanomaterials comprising chiral molecules and nanocrystals: plasmon enhancement, dipole interactions, and dielectric effects. Nano Lett..

[24] Govorov AO (2011). Plasmon-induced circular dichroism of a chiral molecule in the vicinity of metal nanocrystals. Application to various geometries. J. Phys. Chem. C.

[25] Zhang H, Govorov AO (2013). Giant circular dichroism of a molecule in a region of strong plasmon resonances between two neighboring gold nanocrystals. Phys. Rev. B.

[26] Bernal Arango F, Femius Koenderink A (2013). Polarizability tensor retrieval for magnetic and plasmonic antenna design. N. J. Phys..

[27] Bernal Arango F, Coenen T, Femius Koenderink A (2014). Underpinning hybridization intuition for complex nanoantennas by magnetoelectric quadrupolar polarizability retrieval. ACS Photonics.

[28] Caloz C, Sihvola A (2020). Electromagnetic chirality, Part 1: the microscopic perspective [electromagnetic perspectives]. IEEE Antennas Propag. Mag..

[29] Schäferling, M. *Chiral Nanophotonics: Chiral Optical Properties of Plasmonic Systems* (Springer, Cham, 2017).

[30] Zhu AY (2018). Giant intrinsic chiro-optical activity in planar dielectric nanostructures. Light Sci. Appl..

[31] Hu L (2017). Analyzing intrinsic plasmonic chirality by tracking the interplay of electric and magnetic dipole modes. Sci. Rep..

[32] Plum E, Fedotov VA, Zheludev NI (2008). Optical activity in extrinsically chiral metamaterial. Appl. Phys. Lett..

[33] Sersic I (2012). Ubiquity of optical activity in planar metamaterial scatterers. Phys. Rev. Lett..

[34] Kerker M, Wang DS, Giles CL (1983). Electromagnetic scattering by magnetic spheres. J. Opt. Soc. Am..

[35] Liu W, Kivshar YS (2017). Multipolar interference effects in nanophotonics. Philos. Trans. R. Soc. A.

[36] Poutrina E, Urbas A (2014). Multipole analysis of unidirectional light scattering from plasmonic dimers. J. Opt..

[37] Fruhnert M (2017). Computing the T-matrix of a scattering object with multiple plane wave illuminations. Beilstein J. Nanotechnol..

[38] Evlyukhin AB (2012). Demonstration of magnetic dipole resonances of dielectric nanospheres in the visible region. Nano Lett..

[39] Girard C, Dereux A (1996). Near-field optics theories. Rep. Prog. Phys..

[40] Chaumet PC (1998). Evanescent light scattering: the validity of the dipole approximation. Phys. Rev. B.

[41] Mishchenko MI, Travis LD, Mackowski DW (1996). *T*-matrix computations of light scattering by nonspherical particles: a review. J. Quant. Spectrosc. Radiat. Transf..

[42] Fan ZY, Govorov AO (2010). Plasmonic circular dichroism of chiral metal nanoparticle assemblies. Nano Lett..

[43] Fan ZY, Zhang H, Govorov AO (2013). Optical properties of chiral plasmonic tetramers: circular dichroism and multipole effects. J. Phys. Chem. C..

[44] Karst J (2018). Single plasmonic oligomer chiral spectroscopy. Adv. Optical Mater..

[45] Karst J (2019). Chiral scatterometry on chemically synthesized single plasmonic nanoparticles. ACS Nano.

[46] Alaee R, Rockstuhl C, Fernandez-Corbaton I (2019). Exact multipolar decompositions with applications in nanophotonics. Adv. Optical Mater..

[47] Mun J (2020). Describing meta-atoms using the exact higher-order polarizability tensors. ACS Photonics.

[48] Fernandez-Corbaton I, Fruhnert M, Rockstuhl C (2016). Objects of maximum electromagnetic chirality. Phys. Rev. X.

[49] Fernandez-Corbaton I, Fruhnert M, Rockstuhl C (2015). Dual and chiral objects for optical activity in general scattering directions. ACS Photonics.

[50] Fernandez-Corbaton I (2013). Electromagnetic duality symmetry and helicity conservation for the macroscopic Maxwell’S equations. Phys. Rev. Lett..

[51] Prodan E (2003). A hybridization model for the plasmon response of complex nanostructures. Science.

[52] Duan XY, Yue S, Liu N (2015). Understanding complex chiral plasmonics. Nanoscale.

[53] Yin XH (2013). Interpreting chiral nanophotonic spectra: the plasmonic Born-Kuhn model. Nano Lett..

[54] Hentschel M, Ferry VE, Alivisatos AP (2015). Optical rotation reversal in the optical response of chiral plasmonic nanosystems: the role of plasmon hybridization. ACS Photonics.

[55] Lieberman I (2008). Plasmon-resonance-enhanced absorption and circular dichroism. Angew. Chem. Int. Ed..

[56] Maoz BM (2013). Amplification of chiroptical activity of chiral biomolecules by surface plasmons. Nano Lett..

[57] Abdulrahman NA (2012). Induced chirality through electromagnetic coupling between chiral molecular layers and plasmonic nanostructures. Nano Lett..

[58] Lu F (2013). Discrete nanocubes as plasmonic reporters of molecular chirality. Nano Lett..

[59] Ma W (2013). Attomolar DNA detection with chiral nanorod assemblies. Nat. Commun..

[60] Hendry E (2010). Ultrasensitive detection and characterization of biomolecules using superchiral fields. Nat. Nanotechnol..

[61] Zhao Y (2014). Shell-engineered chiroplasmonic assemblies of nanoparticles for zeptomolar DNA detection. Nano Lett..

[62] Zhao Y (2017). Chirality detection of enantiomers using twisted optical metamaterials. Nat. Commun..

[63] Tullius R (2015). “Superchiral” spectroscopy: detection of protein higher order hierarchical structure with chiral plasmonic nanostructures. J. Am. Chem. Soc..

[64] Yoo S, Park QH (2019). Metamaterials and chiral sensing: a review of fundamentals and applications. Nanophotonics.

[65] Novotny, L. & Hecht, B. *Principles of Nano-Optics* 2nd edn. (Cambridge University Press, Cambridge, 2012).

[66] Barron, L. D. *Molecular Light Scattering and Optical Activity* 2nd edn. (Cambridge University Press, Cambridge, 2009).

[67] Wu T, Wang RY, Zhang XD (2015). Plasmon-induced strong interaction between chiral molecules and orbital angular momentum of light. Sci. Rep..

[68] Cameron RP (2014). Discriminatory optical force for chiral molecules. N. J. Phys..

[69] Yang N, Cohen AE (2011). Local geometry of electromagnetic fields and its role in molecular multipole transitions. J. Phys. Chem. B.

[70] Wu T (2017). A giant chiroptical effect caused by the electric quadrupole. Nanoscale.

[71] Mun J, Rho J (2019). Importance of higher-order multipole transitions on chiral nearfield interactions. Nanophotonics.

[72] Nesterov ML (2016). The role of plasmon-generated near fields for enhanced circular dichroism spectroscopy. ACS Photonics.

[73] Lee S, Yoo S, Park QH (2017). Microscopic origin of surface-enhanced circular dichroism. ACS Photonics.

[74] Mun J, Rho J (2018). Surface-enhanced circular dichroism by multipolar radiative coupling. Opt. Lett..

[75] Besteiro LV (2017). Aluminum nanoparticles with hot spots for plasmon-induced circular dichroism of chiral molecules in the UV spectral interval. Adv. Opt. Mater..

[76] Vázquez-Guardado A, Chanda D (2018). Superchiral light generation on degenerate achiral surfaces. Phys. Rev. Lett..

[77] Lipkin DM (1964). Existence of a new conservation law in electromagnetic theory. J. Math. Phys..

[78] Bliokh KY, Nori F (2011). Characterizing optical chirality. Phys. Rev. A.

[79] Coles MM, Andrews DL (2012). Chirality and angular momentum in optical radiation. Phys. Rev..

[80] Poulikakos LV (2016). Optical chirality flux as a useful far-field probe of chiral near fields. ACS Photonics.

[81] Poulikakos LV (2018). Chiral light design and detection inspired by optical antenna theory. Nano Lett..

[82] Vázquez-Lozano JE, Martínez A (2018). Optical chirality in dispersive and lossy media. Phys. Rev. Lett..

[83] Nieto-Vesperinas M (2015). Optical theorem for the conservation of electromagnetic helicity: significance for molecular energy transfer and enantiomeric discrimination by circular dichroism. Phys. Rev. A.

[84] Nieto-Vesperinas M (2017). Chiral optical fields: a unified formulation of helicity scattered from particles and dichroism enhancement. Philos. Trans. R. Soc. A.

[85] Gutsche P, Nieto-Vesperinas M (2018). Optical chirality of time-harmonic wavefields for classification of scatterers. Sci. Rep..

[86] Schäferling M (2012). Tailoring enhanced optical chirality: design principles for chiral plasmonic nanostructures. Phys. Rev. X.

[87] Schäferling M (2014). Helical plasmonic nanostructures as prototypical chiral near-field sources. ACS Photonics.

[88] Schäferling M (2016). Reducing the complexity: enantioselective chiral near-fields by diagonal slit and mirror configuration. ACS Photonics.

[89] Hendry E (2012). Chiral electromagnetic fields generated by arrays of nanoslits. Nano Lett..

[90] Garciá-Guirado J (2018). Enantiomer-selective molecular sensing using racemic nanoplasmonic arrays. Nano Lett..

[91] Schäferling M, Yin XH, Giessen H (2012). Formation of chiral fields in a symmetric environment. Opt. Express.

[92] Davis TJ, Hendry E (2013). Superchiral electromagnetic fields created by surface plasmons in nonchiral metallic nanostructures. Phys. Rev. B.

[93] Hanifeh, M., Albooyeh, M. & Capolino, F. Helicity maximization below the diffraction limit. Preprint at https://arxiv.org/abs/1906.07170 (2019).

[94] Finazzi M (2015). Quasistatic limit for plasmon-enhanced optical chirality. Phys. Rev. B.

[95] García-Etxarri A, Dionne JA (2013). Surface-enhanced circular dichroism spectroscopy mediated by nonchiral nanoantennas. Phys. Rev. B.

[96] Yoo S, Cho M, Park QH (2014). Globally enhanced chiral field generation by negative-index metamaterials. Phys. Rev. B.

[97] Ho CS (2017). Enhancing enantioselective absorption using dielectric nanospheres. ACS Photonics.

[98] Solomon ML (2019). Enantiospecific optical enhancement of chiral sensing and separation with dielectric metasurfaces. ACS Photonics.

[99] Yao K, Liu YM (2018). Enhancing circular dichroism by chiral hotspots in silicon nanocube dimers. Nanoscale.

[100] Mohammadi E (2019). Accessible superchiral near-fields driven by tailored electric and magnetic resonances in all-dielectric nanostructures. ACS Photonics.

[101] Pellegrini G (2017). Chiral surface waves for enhanced circular dichroism. Phys. Rev. B.

[102] Cameron RP, Barnett SM, Yao AM (2012). Optical helicity, optical spin and related quantities in electromagnetic theory. N. J. Phys..

[103] Bliokh KY, Bekshaev AY, Nori F (2013). Dual electromagnetism: helicity, spin, momentum and angular momentum. N. J. Phys..

[104] Cameron RP (2014). On the ‘second potential’ in electrodynamics. J. Opt..

[105] Alpeggiani F (2018). Electromagnetic helicity in complex media. Phys. Rev. Lett..

[106] Crimin F (2019). Optical helicity and chirality: conservation and sources. Appl. Sci..

[107] Zhang QF (2019). Unraveling the origin of chirality from plasmonic nanoparticle-protein complexes. Science.

[108] Yang L (2019). Chiral nanoparticle-induced enantioselective amplification of molecular optical activity. Adv. Funct. Mater..

[109] Sun P (2018). Helical nanoparticle-induced enantiospecific adsorption of N3 dyes. Chem. Commun..

[110] Ashkin A (1986). Observation of a single-beam gradient force optical trap for dielectric particles. Opt. Lett..

[111] Crocker JC (1999). Entropic attraction and repulsion in binary colloids probed with a line optical tweezer. Phys. Rev. Lett..

[112] Reiserer A (2013). Ground-state cooling of a single atom at the center of an optical cavity. Phys. Rev. Lett..

[113] Cecconi G (2005). Direct observation of the three-state folding of a single protein molecule. Science.

[114] Grier DG (2003). A revolution in optical manipulation. Nature.

[115] Li HT (2006). Evidence for resonance optical trapping of individual fluorophore-labeled antibodies using single molecule fluorescence spectroscopy. J. Am. Chem. Soc..

[116] Wen JD (2008). Following translation by single ribosomes one codon at a time. Nature.

[117] Chang DE (2009). Trapping and manipulation of isolated atoms using nanoscale plasmonic structures. Phys. Rev. Lett..

[118] Liu M (2010). Light-driven nanoscale plasmonic motors. Nat. Nanotechnol..

[119] Chen J (2011). Optical pulling force. Nat. Photonics.

[120] Maher-McWilliams C, Douglas P, Barker PF (2012). Laser-driven acceleration of neutral particles. Nat. Photonics.

[121] Roxworthy BJ (2012). Application of plasmonic bowtie nanoantenna arrays for optical trapping, stacking, and sorting. Nano Lett..

[122] Neuman KC, Block SM (2004). Optical trapping. Rev. Sci. Instrum..

[123] Verdeny I (2011). Optical trapping: a review of essential concepts. Óptica Pura y. Aplicada.

[124] Lin Q, Lin HZ (2017). On deriving the Maxwell stress tensor method for calculating the optical force and torque on an object in harmonic electromagnetic fields. Eur. J. Phys..

[125] Griffiths, D. J. *Introduction to Electrodynamics* 4th edn. (Pearson, Boston, 2014).

[126] Jackson, J. D. *Classical Electrodynamics* 3rd edn. (Wiley, Hoboken, 1999).

[127] Rahimzadegan A (2016). Optical force and torque on dipolar dual chiral particles. Phys. Rev. B.

[128] Chen HJ (2017). Optical torque on small chiral particles in generic optical fields. Opt. Express.

[129] Ding K (2014). Realization of optical pulling forces using chirality. Phys. Rev. A.

[130] Almaas E, Brevik I (1995). Radiation forces on a micrometer-sized sphere in an evanescent field. J. Optical Soc. Am. B.

[131] Barton JP, Alexander DR, Schaub SA (1989). Theoretical determination of net radiation force and torque for a spherical particle illuminated by a focused laser beam. J. Appl. Phys..

[132] Arnaut LR (1997). Chirality in multi-dimensional space with application to electromagnetic characterisation of multi-dimensional chiral and semi-chiral media. J. Electromagn. Waves Appl..

[133] Wang SB, Chan CT (2014). Lateral optical force on chiral particles near a surface. Nat. Commun..

[134] Patterson D, Schnell M, Doyle JM (2013). Enantiomer-specific detection of chiral molecules via microwave spectroscopy. Nature.

[135] Smith D (2014). Photophoretic separation of single-walled carbon nanotubes: a novel approach to selective chiral sorting. Phys. Chem. Chem. Phys..

[136] Bliokh KY, Bekshaev AY, Nori F (2014). Extraordinary momentum and spin in evanescent waves. Nat. Commun..

[137] Tkachenko G, Brasselet E (2014). Optofluidic sorting of material chirality by chiral light. Nat. Commun..

[138] Hayat A, Mueller JPB, Capasso F (2015). Lateral chirality-sorting optical forces. Proc. Natl Acad. Sci. USA.

[139] Tkachenko G, Brasselet E (2014). Helicity-dependent three-dimensional optical trapping of chiral microparticles. Nat. Commun..

[140] Allen L (1992). Orbital angular momentum of light and the transformation of Laguerre-Gaussian laser modes. Phys. Rev. A.

[141] Van Enk SJ, Nienhuis G (1994). Commutation rules and eigenvalues of spin and orbital angular momentum of radiation fields. J. Mod. Opt..

[142] Van Enk SJ, Nienhuis G (1994). Spin and orbital angular momentum of photons. Europhys. Lett..

[143] Yílmaz, H. *Introduction to the Theory of Relativity and the Principles of Modern Physics (A Blaisdell book in the Pure and Applied Sciences)* (Blaisdell Pub. Co, New York, 1965).

[144] Jauch, J. M. & Rohrlich, F. *The Theory of Photons and Electrons* (Addison-Wesley, Cambridge, 1955).

[145] Barut, A. O. *Electrodynamics and Classical Theory of Fields and Particles (Dover Books on Physics)* (Dover, New York, 1980).

[146] Cohen-Tannoudji, C., Dupont-Roc, J. & Grynberg, G. Photons & *Atoms* (Wiley, New York, 1997).

[147] Leader E (2013). The angular momentum controversy: what’s it all about and does it matter?. Phys. Part. Nucl..

[148] Dávila Romero LC, Andrews DL, Babiker M (2002). A quantum electrodynamics framework for the nonlinear optics of twisted beams. J. Opt. B.

[149] Loudon R (2003). Theory of the forces exerted by Laguerre-Gaussian light beams on dielectrics. Phys. Rev. A.

[150] Jáuregui R (2004). Rotational effects of twisted light on atoms beyond the paraxial approximation. Phys. Rev. A.

[151] García-García J (2014). Simple technique for generating the perfect optical vortex. Opt. Lett..

[152] Barnett SM (2016). On the natures of the spin and orbital parts of optical angular momentum. J. Opt..

[153] Alexandrescu A, Cojoc D, Fabrizio ED (2006). Mechanism of angular momentum exchange between molecules and Laguerre-Gaussian beams. Phys. Rev. Lett..

[154] Mondal PK, Deb B, Majumder S (2014). Angular momentum transfer in interaction of Laguerre-Gaussian beams with atoms and molecules. Phys. Rev. A.

[155] Babiker M (2002). Orbital angular momentum exchange in the interaction of twisted light with molecules. Phys. Rev. Lett..

[156] Andrews DL, Romero LCD, Babiker M (2004). On optical vortex interactions with chiral matter. Opt. Commun..

[157] Van Veenendaal M, McNulty I (2007). Prediction of strong dichroism induced by X rays carrying orbital momentum. Phys. Rev. Lett..

[158] Araoka F (2005). Interactions of twisted light with chiral molecules: an experimental investigation. Phys. Rev. A.

[159] Löffler W, Broer DJ, Woerdman JP (2011). Circular dichroism of cholesteric polymers and the orbital angular momentum of light. Phys. Rev. A.

[160] Forbes KA, Andrews DL (2018). Optical orbital angular momentum: twisted light and chirality. Opt. Lett..

[161] Reddy IVAK (2018). Interaction of structured light with a chiral plasmonic metasurface: giant enhancement of chiro-optic response. ACS Photonics.

[162] Wang S (2018). Angular momentum-dependent transmission of circularly polarized vortex beams through a plasmonic coaxial nanoring. IEEE Photonics J..

[163] Brullot W (2016). Resolving enantiomers using the optical angular momentum of twisted light. Sci. Adv..

[164] Zambrana-Puyalto X, Vidal X, Molina-Terriza G (2014). Angular momentum-induced circular dichroism in non-chiral nanostructures. Nat. Commun..

[165] Kerber RM (2017). Reading the orbital angular momentum of light using plasmonic nanoantennas. ACS Photonics.

[166] Afanasev A, Carlson CE, Solyanik M (2017). Circular dichroism of twisted photons in non-chiral atomic matter. J. Opt..

[167] Kong XT (2018). Photothermal circular dichroism induced by plasmon resonances in chiral metamaterial absorbers and bolometers. Nano Lett..

[168] Liu TJ (2019). Chiral plasmonic nanocrystals for generation of hot electrons: toward polarization-sensitive photochemistry. Nano Lett..

[169] Han B (2018). Magnetic circular dichroism in nanomaterials: new opportunity in understanding and modulation of excitonic and plasmonic resonances. Adv. Mater..

[170] Chen SM (2019). Strong nonlinear optical activity induced by lattice surface modes on plasmonic metasurface. Nano Lett..

[171] Gui LL (2019). Nonlinear born-kuhn analog for chiral plasmonics. ACS Photonics.

[172] Ma W, Cheng F, Liu YM (2018). Deep-learning-enabled on-demand design of chiral metamaterials. ACS Nano.

[173] So S (2020). Deep learning enabled inverse design in nanophotonics. Nanophotonics.

[174] So S, Rho J (2019). Designing nanophotonic structures using conditional deep convolutional generative adversarial networks. Nanophotonics.

[175] So S, Mun J, Rho J (2019). Simultaneous inverse-design of materials and structures via deep-learning: demonstration of dipole resonance engineering using core-shell nanoparticles. ACS Appl. Mater. Interfaces.

[176] Lee HE (2018). Amino-acid-and peptides-directed synthesis of chiral plasmonic gold nanoparticles. Nature.

[177] Lee HE (2020). Cysteine-encoded chirality evolution in plasmonic rhombic dodecahedral gold nanoparticles. Nat. Commun..

[178] Lee YY (2020). Plasmonic metamaterials for chiral sensing applications. Nanoscale.

[179] Kulkarni C (2020). Highly efficient and tunable filtering of electrons’ spin by supramolecular chirality of nanofiber-based materials. Adv. Mater..

[180] Im SW (2020). Chiral surface and geometry of metal nanocrystals. Adv. Mater..

